# Single-Cell Atlas of Spleen Remodeling Reveals Macrophage Subset-Driven ASFV Pathogenesis

**DOI:** 10.3390/biology14070882

**Published:** 2025-07-18

**Authors:** Liyuan Wang, Shouzhang Sun, Lei Liu, Yun Chen, Haixue Zheng, Zhonglin Tang

**Affiliations:** 1 Shenzhen Branch, Guangdong Laboratory for Lingnan Modern Agriculture, Agricultural Genomics Institute at Shenzhen, Chinese Academy of Agricultural Sciences, Shenzhen 518124, China; 2 Key Laboratory of Livestock and Poultry Multi-Omics of MARA, Agricultural Genomics Institute at Shenzhen, Chinese Academy of Agricultural Sciences, Shenzhen 518124, China; 3 Guangxi Zhuang Autonomous Region Livestock and Poultry Variety Improvement Station, Nanning 530021, China; 4 Yazhouwan National Laboratory, Sanya 572024, China; 5 Lanzhou Veterinary Research Institute, Chinese Academy of Agricultural Sciences, Lanzhou 730046, China; 6 African Swine Fever Regional Laboratory of China, Lanzhou Veterinary Research Institute, Chinese Academy of Agricultural Sciences, Lanzhou 730046, China; 7 Gansu Province Research Center for Basic Disciplines of Pathogen Biology, Lanzhou 730046, China; 8 Kunpeng Institute of Modern Agriculture at Foshan, Agricultural Genomics Institute at Shenzhen, Chinese Academy of Agricultural Sciences, Foshan 528226, China

**Keywords:** ASFV, scRNA-seq, macrophage subsets, viral pathogenesis

## Abstract

African swine fever virus (ASFV) causes global swine outbreaks, but its cellular pathogenesis is poorly understood. Using single-cell RNA data from ASFV-infected pig spleens across four timepoints, we identified macrophages as the primary viral reservoir, with infection driving lymphoid depletion and myeloid expansion. We characterized four functionally distinct macrophage subsets, including a metabolically reprogrammed SusceptibleMac population serving as the major viral niche and an AntiviralMac subset rapidly depleted during infection. Viral gene expression analysis revealed E165R as a central hub in viral replication networks, while host transcriptomics uncovered disruption of Netrin signaling pathways that may facilitate immune evasion. Pseudotime analysis revealed dynamic macrophage state transitions during infection. These findings provide a high-resolution cellular atlas of ASFV pathogenesis, revealing macrophage subset-specific responses that shape disease outcomes and identifying potential targets for therapeutic intervention.

## Simple Summary

African swine fever virus (ASFV) is a deadly pig disease with poorly understood cellular mechanisms. Using single-cell technology, we analyzed infected pig spleens and found macrophages—a type of immune cell—to be the virus’s primary target. Infection depletes disease-fighting cells while favoring virus-supportive conditions. We identified four macrophage subtypes, including one highly susceptible to infection and another with antiviral properties that is rapidly destroyed. The viral E165R gene was essential for ASFV replication while disrupting host defense signals. By tracking cellular changes, we revealed how macrophages drive ASFV progression. Our findings offer insights for future therapies against this global threat.

## 1. Introduction

African swine fever (ASF) is a highly contagious and lethal pathogen affecting domestic pigs and wild boars, causing severe economic losses to the global swine industry [1]. African swine fever virus (ASFV) is a large, double-stranded DNA virus and the sole member of the *Asfarviridae* family, encoding over 150 proteins, many of which directly interfere with host gene expression, immune signaling, and metabolic pathways [2]. ASFV replicates exclusively in the cytoplasm of infected cells, forming large perinuclear viral factories near the microtubule organizing center. These factories serve as sites for viral genome replication, transcription, and particle assembly, while simultaneously recruiting host organelles (e.g., mitochondria, ribosomes) and modulating cellular processes to support viral propagation [3]. The virus primarily targets cells of the mononuclear phagocyte system, particularly macrophages, which serve as the main site for viral replication and dissemination [4,5]. Notably, the formation of viral factories in infected macrophages may induce profound transcriptional and metabolic reprogramming, potentially explaining observed shifts in host gene expression profiles during infection. ASFV exhibits complexity due to its large genome, encoding multifunctional proteins sufficient for productive replication [6]. Despite extensive research, the mechanisms underlying ASFV tropism, host immune evasion, and disease progression remain incompletely understood [7], hindering the development of effective vaccines and antiviral therapies.

The spleen plays a crucial role in ASFV pathogenesis, exhibiting the highest viral DNA load among infected tissues, severe histopathological lesions, and serving as a major site for viral replication and immune disruption [8,9]. Previous bulk transcriptomic and histopathological studies documented severe lymphoid depletion and macrophage hyperplasia in ASFV-infected spleens [10,11], suggesting profound immune dysregulation. However, these approaches lack the resolution to dissect the heterogeneity of immune cell responses at the single-cell level, obscuring key cellular subsets that may drive infection outcomes. Recent advances in single-cell RNA sequencing (scRNA-seq) revolutionized our ability to characterize complex host–pathogen interactions, enabling the identification of rare cell populations, dynamic transcriptional states, and cell-specific viral tropism. While Zheng et al. characterized the single-cell transcriptomic landscape of ASFV-infected porcine alveolar macrophages, revealing broad host–pathogen interplay, their analysis did not address tissue-specific responses in major viral replication sites like the spleen [12]. Subsequent work by Zhu et al. identified a spleen-specific shift in ASFV tropism from macrophages to CD14-negative immature monocytes using scRNA-seq [9]. However, this analysis focused primarily on monocyte shifts without resolving functionally distinct macrophage subpopulations that may drive early infection dynamics.

ASFV disrupts macrophage function through unclear understood mechanisms [12]. Emerging evidence suggests that viruses often hijack conserved axon guidance pathways—such as Netrin signaling—to manipulate immune cells [13]. Netrin-1 (NTN1) and its receptors (e.g., DCC, UNC5B, NEO1) are traditionally associated with neuronal development but are now recognized as critical regulators of macrophage behavior [14,15]. Netrin-1, a secreted laminin-like protein, critically regulates macrophage behavior in both acute and chronic inflammation [14]. In acute infection, Netrin-1 directs monocyte/macrophage migration to inflamed tissues, while in chronic settings (e.g., atherosclerosis), it traps macrophages by suppressing their chemotactic egress [16]. This dual role makes Netrin signaling a plausible target for viral subversion. Although Netrin-1’s involvement in bacterial and sterile inflammation is established, its interaction with viral infections—particularly ASFV—remains unexplored. Given ASFV’s reliance on macrophages for dissemination, we hypothesized that the virus might disrupt Netrin-mediated communication to impair macrophage mobility or function.

In this study, we analyzed spleen scRNA-seq data from ASFV-infected pigs across four time points (Days 0, 3, 5, and 7 post-challenge) to systematically map the immune landscape during infection. Our objectives were as follows: (1) define the dynamic changes in spleen immune cell composition and identify key cell populations affected by ASFV infection; (2) characterize viral tropism at single-cell resolution, determining which subsets are permissive to infection and how viral load correlates with host gene expression; (3) investigate macrophage heterogeneity, uncovering functionally distinct subpopulations with varying susceptibility to ASFV and their roles in viral replication and immune evasion; and (4) elucidate transcriptional and intercellular communication networks that are disrupted during infection, providing insights into potential mechanisms of immune suppression.

Our findings revealed that ASFV infection drives a dramatic shift in the spleen immune microenvironment, marked by the depletion of adaptive immune cells (B and T cells) and the expansion of myeloid subsets, particularly macrophages. We identified a highly susceptible macrophage subpopulation (SusceptibleMac) that serves as the primary viral reservoir, exhibiting progressive infection and metabolic reprogramming to support viral replication. In contrast, an antiviral macrophage subset (AntiviralMac) is rapidly depleted, suggesting targeted elimination by the virus. Furthermore, we uncovered significant disruptions in intercellular communication, including the downregulation of Netrin signaling, which may contribute to immune dysregulation during infection. By integrating temporal, transcriptional and functional analyses, this study provided a comprehensive atlas of ASFV-host interactions in the spleen, offering new mechanistic insights into viral pathogenesis. These findings not only enhanced our understanding of ASFV immunobiology but also highlighted potential therapeutic targets for modulating macrophage responses and improving antiviral strategies.

## 2. Materials and Methods

### 2.1. scRNA-Seq Data Acquisition and Quality Control

Thirty-three specific-pathogen-free pigs (10 weeks old, ~40 kg) were screened negative for ASFV and 7 common swine viruses (from Zhu et al. PNAS 2024) [9]. The animals were inoculated intramuscularly with the indicated amounts of ASFV CN/GS/2018 or equal amounts of solvent control, while 3 controls received solvent only. Animals were housed in BSL-3 facilities with daily veterinary monitoring. Spleens were dissected at necropsy (prioritized for scRNA-seq based on Zhu et al.’s findings of high viral load [9]). Single-cell suspensions were processed using 10× Genomics Chromium (v3 chemistry, Pleasanton, CA, USA). Data were analyzed with Cell Ranger (v6.1.2).

The scRNA-seq data were downloaded from the GEO database with accession number PRJNA879060 [9]. The ASFV reference genome data were downloaded from NCBI via data from the WUHAN strain (MN393477.1 [17]). A mixed genome reference was first constructed by Cell Ranger 8.0.0 (10× Genomics, Pleasanton, CA, USA) cellranger mkref to process the raw sequencing data of scRNA-seq. Subsequent dimensionality reduction and clustering analyses were conducted via the Seurat package (version 5.0.1) [18]. Initially, genes expressed in fewer than three cells per sample were filtered out. To minimize potential interference from hematopoietic cells in spleen tissue, we excluded cells expressing hemoglobin-related genes. Cells with more than 1% of their transcripts mapping to these hemoglobin-related genes were filtered out using the subset function. Low-quality cells were removed on the percentage of detected mitochondrial genes (percentage of MT) less than 10% and ambient RNAs contamination less than 0.2 [19].

### 2.2. scRNA Data Processing and Annotation

Following quality control, all samples were normalized using the normalizeData function to standardize expression values across cells in R (4.4.2). Gene expression was then scaled using ScaleData to mitigate technical artifacts and biological heterogeneity. Highly variable genes (n = 2000) were selected via FindVariableFeatures (method = “vst”) for downstream analyses in Seurat (5.0.1). Principal component analysis (PCA) was performed on these genes using RunPCA, followed by batch correction with Harmony (v1.2.0) to integrate multi-sample datasets. Cell clustering was conducted by building a K-nearest neighbor graph (KNN, FindNeighbors) on the first 20 Harmony-adjusted PCs and applying Leiden clustering (FindClusters, resolution = 0.01–0.8). Two-dimensional embeddings were generated using UMAP (RunUMAP) for visualization. Cell populations were annotated through a multi-step approach. First, cluster-specific marker genes were identified using FindAllMarkers. Automated cell type prediction was performed with easybio package (v1.1.1) [20]. Annotations were further validated against the CellMarker 2.0 database (http://xteam.xbio.top/CellMarker/, accessed on 12 June 2025) to ensure biological relevance. We identified ASFV RNA in single cells using aligned reads matching the ASFV genome (GenBank: MN393477.1). Cells with ≥1 viral UMI were considered viral fragment-containing. Viral load was calculated as the percentage of total UMIs mapping to ASFV. To distinguish true infection from ambient RNA, we applied Otsu’s thresholding to log-transformed viral UMI counts using a custom R script [9], with infection thresholds determined separately for each sample (Figure S1b,c).

### 2.3. Differential Expression and Functional Enrichment

Differentially expressed genes (DEGs) between conditions were detected using FindMarkers in Seurat (5.0.1), with thresholds of “min.pct = 0.25, logfc.threshold = 0.25”. DEGs heatmapping and pathway enrichment analysis was conducted via ClusterGVis (package version: 0.1.2, https://github.com/junjunlab/ClusterGVis, accessed on 15 February 2025).

### 2.4. Cell–Cell Communication Inference

We analyzed cell–cell communication dynamics using CellChat (v2.1.2) [21] with its Secreted Signaling database (CellChatDB.human), performing independent probabilistic modeling of ligand–receptor interactions for each time point (Days 0, 3, 5, and 7) to ensure biological plausibility of coexisting cell populations. Network centrality metrics identified key pathways within each temporal group, with subsequent comparative analysis revealing infection-stage-specific communication patterns (e.g., early inflammatory vs. late immunosuppressive signaling) without assuming direct cross-timepoint interactions. All predicted ligand–receptor pairs were validated against established macrophage-lymphocyte crosstalk mechanisms, and results were visualized using CellChat’s integrated tools to highlight temporal evolution of communication potential.

### 2.5. Transcription Factor Network Reconstruction

Regulatory networks were inferred via pySCENIC (v0.12.1) [22]. Co-expression modules were derived using GRNBoost, followed by motif enrichment (RcisTarget) to link transcription factors (TFs) to target genes. TF activity scores were calculated with AUCell and visualized as networks (igraph, v1.6.0).

### 2.6. Pseudotime Trajectory Analysis

The cell lineage trajectory was inferred using Monocle 2 (2.34.0) according to the tutorial. After the cell trajectory was constructed, DDRtree (0.1.5) was used to visualize it in two-dimensional space. The dynamic expression changes of selected marker genes by pseudotime were visualized by ClusterGVis (package version: 0.1.2, https://github.com/junjunlab/ClusterGVis, accessed on 15 February, 2025).

### 2.7. Statistical Analysis for Differential Expression

Differential gene expression analysis was performed using the Wilcoxon rank-sum test implemented in Seurat’s FindMarkers(5.0.1) function. Genes were considered significantly differentially expressed if they met dual thresholds: (1) adjusted *p*-value < 0.05 (Benjamini–Hochberg correction) and (2) absolute log2 fold change > 0.25. These parameters were uniformly applied across all cell type comparisons to ensure consistency in identifying biologically relevant changes while controlling for multiple testing. The log2FC threshold of 0.25 (~20% expression change) was selected to capture subtle but potentially important transcriptional alterations during early infection stages.

## 3. Results

### 3.1. Dynamic Change in Immune Cell Composition in the Spleen Following ASFV Infection

**Figure 1:**
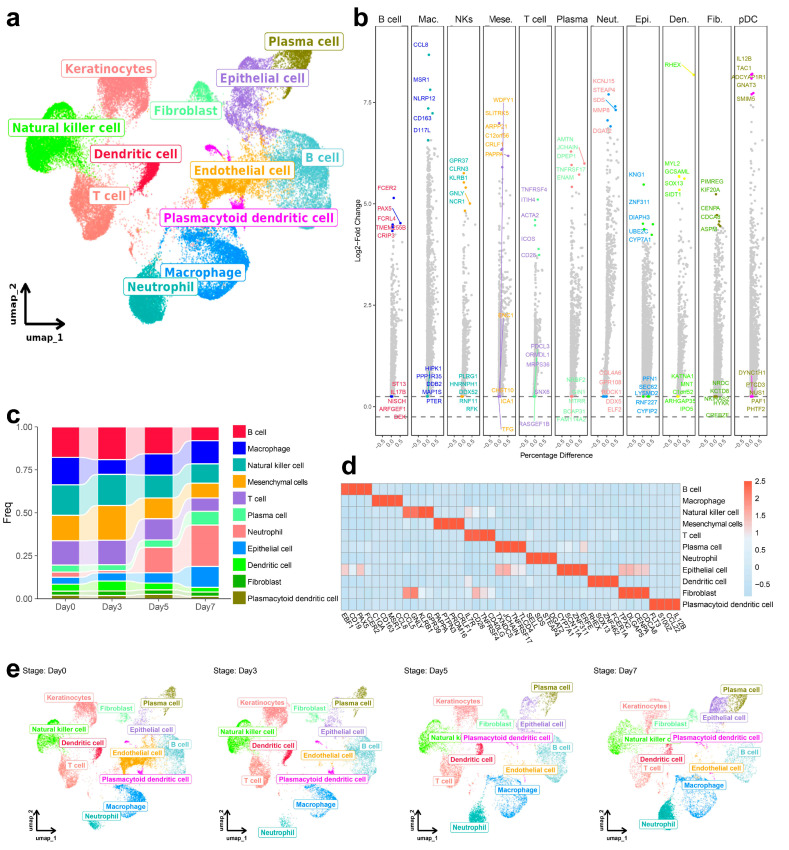
Single-cell transcriptomic landscape of spleen cells across four time points following ASFV infection. (**a**) UMAP visualization of spleen cells collected at four time points post-challenge (Day 0, 3, 5, and 7), colored by cell type. Distinct immune populations were identified based on canonical marker genes and unsupervised clustering. (**b**) Volcano plots showing differentially expressed genes (DEGs) for each major cell type upon ASFV infection. For each comparison, DEGs were identified using the Wilcoxon rank-sum test. Genes with an adjusted *p*-value < 0.05 and logfc.threshold = 0.25. (**c**) Stacked line-bar plots depicting dynamic changes in the abundance of each cell type over the infection course. Relative frequencies (shown as percentages of total spleen cells) are presented for each time point. (**d**) Heatmap displaying the expression patterns of representative marker genes across identified cell clusters. The scale represents normalized expression levels (Z-scores), allowing comparison across genes and cell types. (**e**) Time-course visualization of spleen cells.

Single-cell RNA sequencing of spleen from ASFV-infected pigs (Days 0, 3, 5, and 7 post-challenge) yielded 49,886 high-quality cells after quality control and sample integration. Unsupervised clustering and UMAP dimensionality reduction demonstrated distinct cell population segregation (Figure 1a), enabling the identification of 11 major immune and stromal cell types based on canonical marker gene expression: B cells (CD19^+^), T cells (IL7R^+^), macrophages (CD163^+^), neutrophils (SELL^+^), dendritic cells (FCER1A^+^), NK cells (GNLY^+^), plasma cells (JCHAIN^+^), epithelial cells (SCN1A^+^), fibroblasts (TPX2^+^), mesenchymal cells (PAPPA^+^), and Plasmacytoid dendritic cells (S100Z^+^) (Figure 1b,d). Viral gene expression analysis revealed selective enrichment of ASFV gene D117L in macrophages, consistent with established ASFV cellular tropism [23]. This viral gene emerged as a dominant marker in macrophage clusters, confirming their role as primary viral reservoirs during infection (Figure 1b). Conversely, B cells exhibited robust PAX5 expression without detectable ASFV gene expression.

Temporal analysis of cellular composition using stacked line-bar plots (Figure 1c,e) revealed progressive decline in lymphoid populations, including B and T cells, throughout infection (Figure S1a). Simultaneously, myeloid compartments demonstrated increased abundance, with macrophages expanding significantly while neutrophils exhibited a steady upward trend across the infection timeline. These findings demonstrated that ASFV infection drives substantial remodeling of the spleen immune microenvironment, characterized by depletion of adaptive immune cell populations concurrent with expansion of innate immune cells, particularly macrophages which serve as primary targets for viral infection.

### 3.2. ASFV Preferentially Infects Macrophages with Increasing Viral Loads and Transcriptional Reprogramming over Time

**Figure 2:**
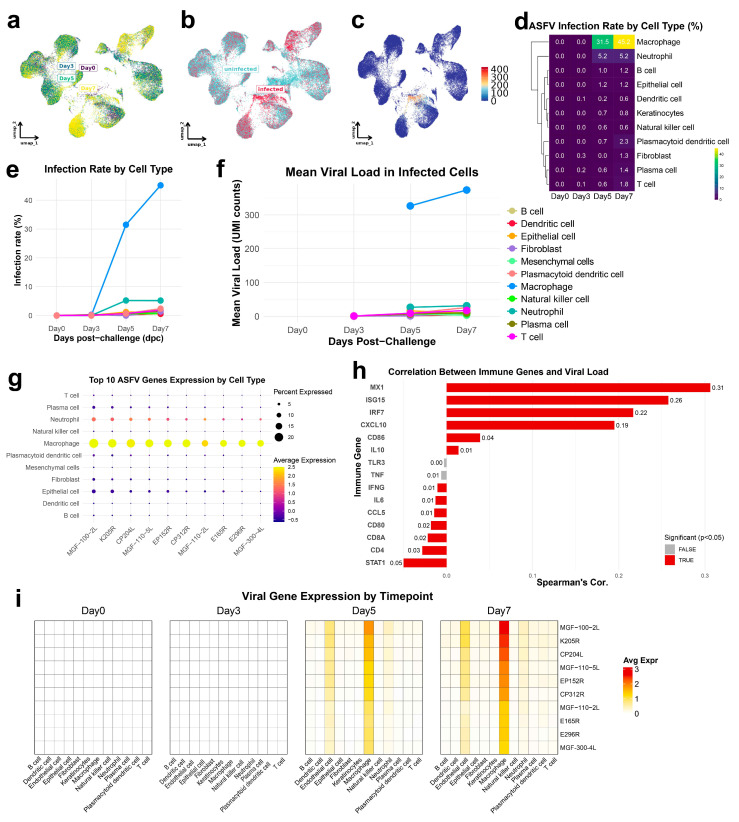
Viral dynamics and cell-type-specific ASFV expression across different time points post-challenge. (**a**) UMAP visualization showing the distribution of spleen cells at different time points (Day 0, 3, 5, and 7) post-challenge. (**b**) UMAP plot colored by infection status (infected vs. ininfected). Cells are clustered by gene expression profile. (**c**) UMAP plot showing the distribution of spleen cells colored by ASFV viral gene expression levels. (**d**) Heatmap of ASFV infection rates across various cell types at different time points. Infection rates were calculated as the percentage of infected cells within each cell type, with values scaled from 0 to 100. (**e**) Line plot showing the infection rates of each cell type over time. (**f**) Line plot displaying the mean viral load in infected cells of each cell type over the course of ASFV infection. (**g**) Bar plot of the top 10 ASFV genes expressed in each cell type, with circle size representing the percentage of expressing cells and color intensity representing the average expression level of each gene. (**h**) Correlation analysis between host gene expression and viral load in infected cells. Interferon_Response (MX1, ISG15, IFIT1, IFIT3, OAS1, STAT1, IRF7, IRF9), Inflammatory (TNF, IL6, IL1B, IL18, CCL2, CXCL8, CXCL10), Anti-inflammatory (IL10, TGFB1, SOCS1, SOCS3), Antigen_Presentation (CD80, CD86, CD40, HLA-DRA, HLA-DRB1), Tcell_Markers (CD3D, CD3E, CD4, CD8A, CD8B, FOXP3, GZMB), PRR, Pattern Recognition Receptor (TLR3, TLR7, TLR8, DDX58, IFIH1, NLRP3). (**i**) Heatmap of the expression patterns of the top ASFV genes across different cell types at different time points.

Single-cell analysis of ASFV gene distribution revealed distinct cell-type tropism, with viral transcripts predominantly localized to macrophage clusters, particularly at later infection stages (Day 5 and Day 7) (Figure 2a,b). Spatiotemporal mapping of viral expression titers across the cellular landscape confirmed macrophages as the primary ASFV reservoir, demonstrating progressive viral load accumulation from Day 3 to Day 7 (Figure 2c and Figure S2a,b). This infection pattern was largely absent in other immune cell populations, highlighting pronounced cell-type specificity in viral replication dynamics.

Quantitative assessment of infection rates demonstrated that macrophages consistently exhibited the highest percentage of ASFV-positive cells across all time points, as visualized in both heatmap and line plot representations (Figure 2d,e). By Day 7 post-infection, a substantial macrophage fraction harbored viral transcripts, while B cells, T cells, and dendritic cells showed minimal-to-moderate infection levels. Analysis of mean viral load within infected cells (Figure 2f) revealed parallel patterns, with macrophages maintaining significantly higher viral transcript levels than other cell types, further establishing their role as the preferential site for viral replication. Comprehensive profiling of viral gene expression demonstrated that key ASFV genes, including MGF-100-2L, K205R, and CP204L, were selectively and robustly expressed in macrophages compared to other immune populations. Early genes (CP204L, E165R) dominate Day 3, while structural genes (K205R [24], B646L) peak at Days 5–7; furthermore, immune evasion genes (MGF-100-2L [25], EP402R) are macrophage-enriched, aligning with ASFV tropism (Figure 2g and Figure S2c). The dot plot visualization confirmed that macrophages clusters exhibited both elevated proportions of viral gene-expressing cells (large dot sizes) and higher average expression levels (intense color saturation).

Correlation analyses between viral load and host gene expression in infected cells revealed significant transcriptional reprogramming (Figure 2h and Figure S3). Multiple macrophage-associated antiviral response genes, including MX1, ISG15, IRF7, CXCL10, CD86, and IL10, demonstrated strong positive correlations with ASFV burden, indicating robust type I interferon-mediated antiviral responses concurrent with active viral replication. Conversely, immune regulatory and lymphocyte-associated genes, such as STAT1, CD4, CD8A, CD80, CCL5, and IL6, showed negative correlations with viral load, potentially reflecting immune suppression or functional alterations within heavily infected macrophages. Temporal analysis of ASFV gene expression across cell types (Figure 2i) further confirmed the selective nature of infection progression. While sporadic viral gene expression appeared in rare plasma and dendritic cells by Day 3, ASFV genes were predominantly and intensely expressed in macrophages by Days 5 and 7, underscoring progressive viral expansion throughout the macrophage compartment.

These findings established macrophages as the predominant cellular targets of ASFV within spleen tissue, exhibiting maximal infection rates, viral loads, and viral gene expression. The observed association between viral burden and macrophage transcriptional signatures suggested that these cells not only support viral replication but undergo extensive immunomodulatory reprogramming during infection. These observations provided a cellular foundation for further mechanistic investigations into macrophage contributions to ASFV pathogenesis.

### 3.3. Subclustering Analysis of Macrophages Reveals Distinct Functional States and Susceptibility to ASFV Infection

**Figure 3:**
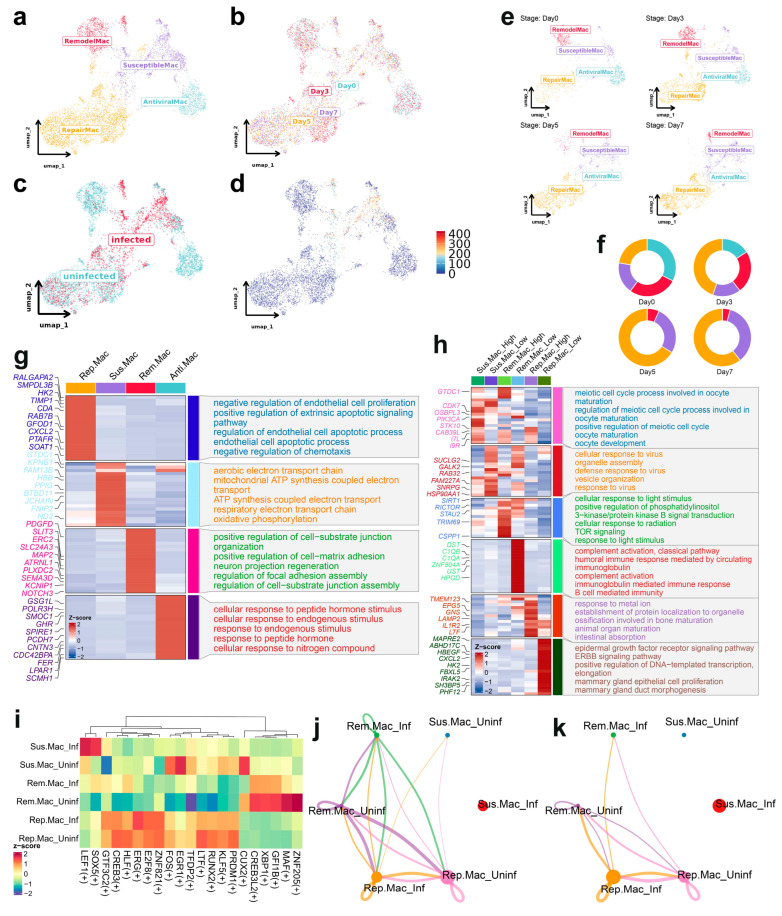
Macrophage subpopulation dynamics during ASFV infection. (**a**) UMAP visualization of spleen macrophages isolated from the integrated single-cell RNA-seq dataset, clustered into four transcriptionally distinct subpopulations: Remodeling Macrophages (RemodelMac, crimson), Susceptible Macrophages (SusceptibleMac, medium purple), Repairing Macrophages (RepairMac, orange), and Antiviral Macrophages (AntiviralMac, cadet Blue). Subcluster identities were determined based on differential gene expression profiles and functional annotation. (**b**) UMAP plots depicting the temporal distribution of macrophage subtypes across the four time points (Day 0, 3, 5, and 7 post-infection). (**c**) UMAP visualization of macrophage subtypes colored by infection status (ASFV-infected vs. uninfected). (**d**) UMAP plot colored by viral load (ASFV gene expression level) among macrophage subtypes. (**e**) UMAP plots depicting the temporal distribution of macrophage subtypes across the four time points (Day 0, 3, 5, and 7 post-infection), respectively. (**f**) Donut charts illustrating the relative abundance of each macrophage subtype at different time points. (**g**) Heatmap of signature gene expression and enriched biological pathways for each macrophage subtype. (**h**) Heatmap displaying the functional enrichment profiles of macrophage subpopulations stratified by viral load. Cells were divided into “high” and “low” viral load groups within each macrophage subtype (RemodelMac, SusceptibleMac, and RepairMac), based on ASFV gene expression levels. (**i**) Heatmap showing the enrichment of transcription factor (TF) regulatory signatures in macrophage subtypes before and after ASFV infection. (**j**,**k**) Cell–cell communication networks between macrophage subtypes during ASFV infection at Day 5 and Day 7, inferred using ligand–receptor interaction analysis.

High-resolution subclustering of spleen macrophages identified four functionally distinct subsets with varying responses to ASFV infection (Figure 3a). The UMAP visualization demonstrated substantial temporal redistribution of these subpopulations across infection timepoints (Figure 3b,c), with SusceptibleMac emerging as the predominant viral reservoir, as evidenced by near-exclusive colocalization with high viral loads (Figure 3d) and progressive expansion from 17.72% (Day 0) to 34.97% (Day 7) of total macrophages (Figure 3e,f). These findings established SusceptibleMac as the principal macrophage subpopulation supporting ASFV replication. Notably, the AntiviralMac subset demonstrated marked depletion, declining from 32.57% (Day 0) to virtual absence by Day 5 (0.14%) and Day7 (0.12%), suggesting active viral suppression of this protective population (Figure 3f). Analysis of ASFV gene expression patterns revealed subpopulation-specific tropism, with most antigen genes predominantly expressed in SusceptibleMac, while MGF genes were primarily detected in RemodelMac cells (Figure S4).

Pathway enrichment analysis revealed distinct functional specializations among macrophage subsets (Figure 3g). RepairMac demonstrated significant enrichment in endothelial cell proliferation and apoptosis regulation pathways, consistent with tissue repair and inflammation control functions. SusceptibleMac exhibited marked enrichment in mitochondrial ATP synthesis and oxidative phosphorylation pathways, suggesting elevated energy requirements to support viral replication. RemodelMac showed enrichment in cell adhesion and neuronal regeneration pathways, indicating roles in tissue remodeling and intercellular communication. AntiviralMac was predominantly associated with cellular response pathways to endogenous stimuli, including peptide hormones, reflecting immunomodulatory functions during early infection. Stratification by viral load revealed further functional partitioning (Figure 3h). SusceptibleMac_High exhibited enrichment in cell cycle and oocyte maturation processes, suggesting proliferative activation during infection, while SusceptibleMac_Low showed enrichment in viral defense pathways, including viral response and organelle assembly. Within RemodelMac, high viral loads correlated with cellular responses to light stimuli and TOR signaling, while low viral loads associated with complement activation and B cell-mediated immunity. RepairMac with high viral loads demonstrated enrichment in metal ion response and organ maturation pathways, whereas low viral load RepairMac cells exhibited association with epidermal growth factor signaling and transcription regulation processes, further supporting their role in tissue restoration and immune regulation.

Transcription factor analysis revealed subpopulation-specific regulatory networks before and after infection (Figure 3i). SusceptibleMac specifically upregulated pro-viral factors LEF1 and SOX5, while RepairMac maintained stable transcription factor networks despite infection. Cell–cell communication analysis at Days 5 and 7 post-infection (Figure 3j,k) revealed progressive communicative isolation of SusceptibleMac, with minimal intercellular signaling by Day 5 and complete absence of detectable communication by Day 7, suggesting functional segregation during advanced infection stages, potentially reflecting disruption of immune coordination networks.

These findings characterized the dynamic alterations in macrophage subpopulations during ASFV infection, highlighting the critical role of SusceptibleMac in viral propagation and the transient but essential contribution of AntiviralMac to early immune defense. Furthermore, the data established RepairMac as a key mediator of tissue repair and inflammation regulation, while identifying RemodelMac as a contributor to structural remodeling of infected tissues. Collectively, these observations provided novel insights into macrophage-mediated immune responses during ASFV infection and underscore the complex immunological dynamics governing disease progression.

### 3.4. Infection-Phase-Specific Transcriptional Programs in Macrophage Subpopulations

**Figure 4:**
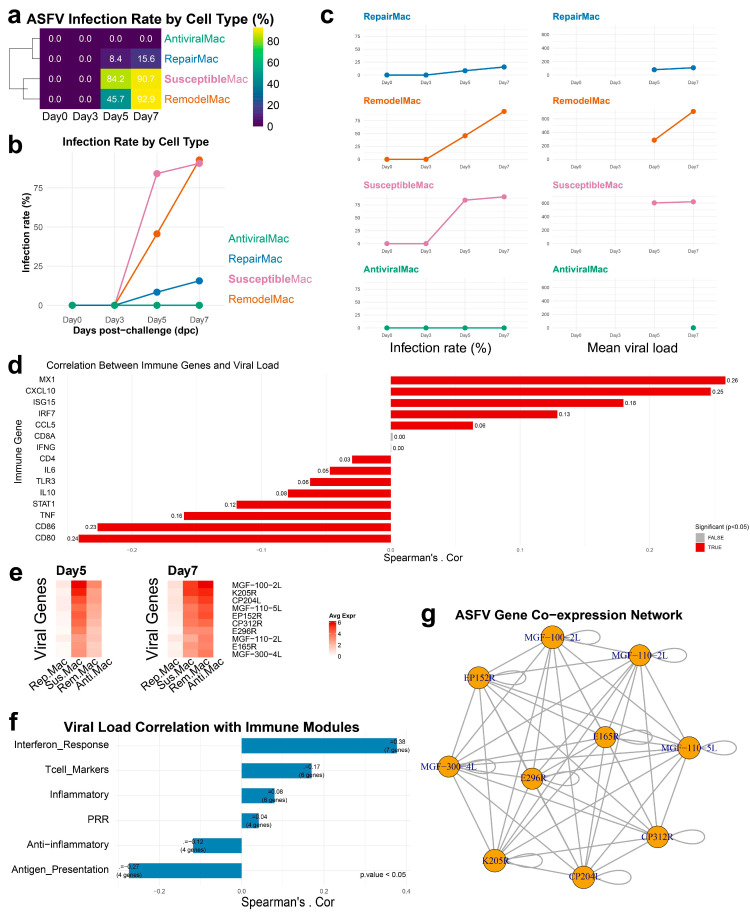
Viral dynamics in sub-macrophages cell types across different time points post-challenge. (**a**) Heatmap of ASFV infection rates across sub-macrophage cell types at different time points. Infection rates were calculated as the percentage of infected cells within each cell type, with values scaled from 0 to 100%. (**b**,**c**) Line plot showing the infection rates of each sub-macrophage cell type over time. (**d**) Correlation analysis between host gene expression and viral load in infected cells. (**e**) Heatmap of the expression patterns of the top ASFV genes across different cell types at different time points. (**f**) Correlation analysis between host gene module expression and viral load in infected cells. Interferon_Response (MX1, ISG15, IFIT1, IFIT3, OAS1, STAT1, IRF7, IRF9), Inflammatory (TNF, IL6, IL1B, IL18, CCL2, CXCL8, CXCL10), Anti-inflammatory (IL10, TGFB1, SOCS1, SOCS3), Antigen_Presentation (CD80, CD86, CD40, HLA-DRA, HLA-DRB1), Tcell_Markers (CD3D, CD3E, CD4, CD8A, CD8B, FOXP3, GZMB), PRR, Pattern Recognition Receptor (TLR3, TLR7, TLR8, DDX58, IFIH1, NLRP3). (**g**) Co-expression analysis of ASFV genes.

Temporal analysis of ASFV infection dynamics across sub-macrophage populations revealed distinct patterns in viral suscetibility, replication efficiency and transcriptional responses. Quantitative assessment of infection rates demonstrated that SusceptibleMac and RemodelMac subpopulations exhibited remarkably high infection rates, exceeding 95% across all examined timepoints (Figure 4a–c). These two subsets displayed significantly elevated susceptibility to ASFV compared to other sub-macrophage populations. Parallel analysis of mean viral burden within infected cells (Figure 4d) confirmed that both SusceptibleMac and RemodelMac maintained substantially higher viral loads throughout infection progression, further establishing their role as primary viral replication reservoirs. Differential transcriptomic analysis between SusceptibleMac, RemodelMac, and RepairMac during late infection stages revealed significant enrichment of ATP synthesis and oxidative phosphorylation pathways (Figure S5), suggesting metabolic reprogramming to support viral replication.

Longitudinal examination of ASFV gene expression profiles revealed a notable transition in viral tropism between Days 5 and 7, with predominant viral gene expression shifting from SusceptibleMac to RemodelMac subpopulations (Figure 4f). This temporal redistribution suggested dynamic alterations in cellular permissiveness factors and immune modulation influence viral targeting during disease progression. Correlation analysis between host gene expression and viral burden identified multiple significant associated functional pathways. Strong positive correlations were observed between viral load and genes associated with interferon response (MX1, ISG15, IFIT1, OAS1, STAT1, IRF7), inflammatory signaling (TNF, IL6, IL1B, CCL2, CXCL10), and pattern recognition receptors (TLR3, TLR7, NLRP3) (Figure 4g). Conversely, genes involved in anti-inflammatory mechanisms (IL10, TGFB1, SOCS1), antigen presentation (CD80, CD86, HLA-DRB1), and T cell markers (CD3D, CD4, CD8A) demonstrated negative correlations with viral burden. Notably, CD86 exhibited divergent correlation patterns between macrophages and the overall cellular population, displaying negative correlation specifically within macrophages despite positive correlation across total cells, indicating macrophage-specific regulatory mechanisms during infection.

Co-expression network analysis of ASFV genes identified E165R-(k1R) and E296R-(k4R) as central nodes within the viral gene interaction network (Figure 4g). Although E165R encodes a functional dUTPase, recent studies demonstrate that its deletion does not impair viral replication in swine macrophages or virulence in vivo [26]. Its network centrality may reflect roles in maintaining genomic fidelity during infection rather than directly driving replication, suggesting alternative mechanisms for its co-option during ASFV infection.

### 3.5. Pseudotemporal Analysis of Macrophage Subtypes During ASFV Infection

**Figure 5:**
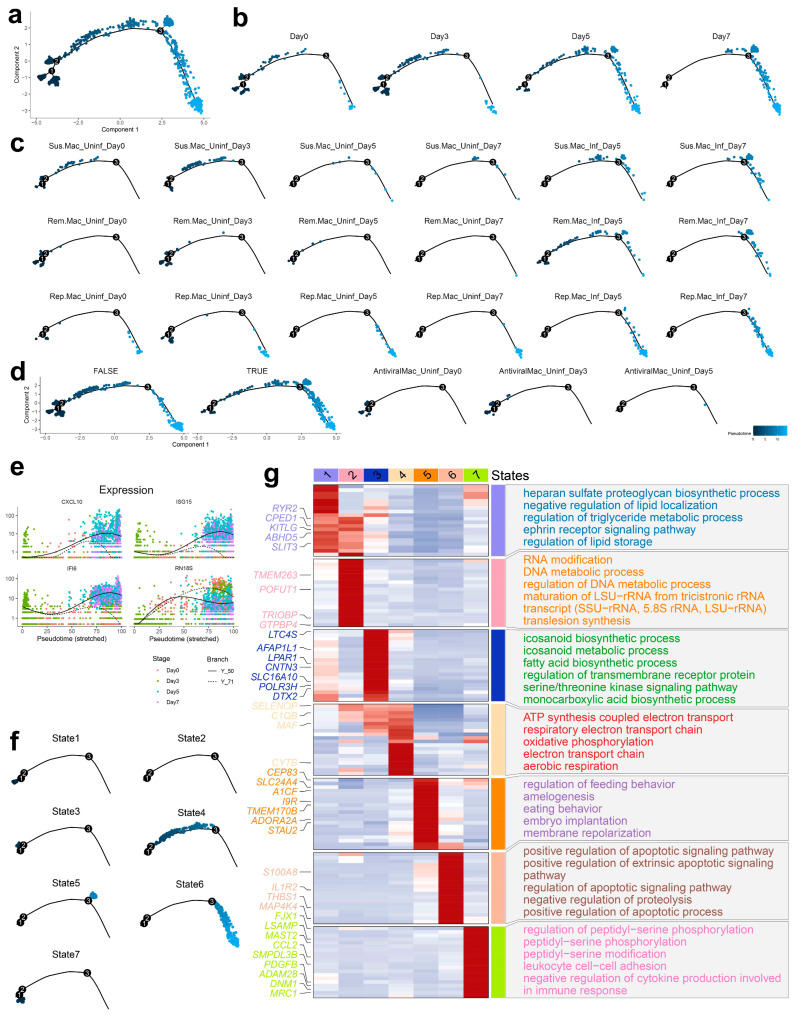
Pseudotemporal analysis of macrophage subtypes. (**a**) Overall trajectory of macrophage subtypes in pseudotime. Black circles with numbers represent trajectory nodes inferred by Monocle2 pseudotime analysis, where higher values indicate later developmental stages. (**b**) Trajectory plot showing the distribution of macrophage subtypes across pseudotime intervals, highlighting the temporal shifts in infection patterns. (**c**) Detailed trajectory plots for each macrophage subtype before and after ASFV infection, revealing subtype-specific changes in infection dynamics over time. (**d**) Comparison of macrophage trajectories before and after ASFV infection, showing the overall impact of infection on the progression of each subtype. (**e**) Expressing changes in marker genes during ASFV infection. (**f**) Distribution of different cell states across the pseudotemporal trajectory. States 1–3 represent macrophage populations at early infection stages. State 4 specifically marks the SusceptibleMac subset unique to early infection. States 5–6 correspond to late-stage infected macrophages. (**g**) Marker genes and enriched pathways for different cell states identified through pseudotemporal analysis.

Pseudotemporal trajectory analysis of macrophage subpopulations revealed distinct developmental continua and transcriptional transitions during ASFV infection progression (Figure 5a). Spatiotemporal mapping of macrophage subtypes across pseudotime intervals demonstrated significant shifts in infection patterns, with subtype-specific transitions from uninfected to infected states (Figure 5b,d). Granular examination of individual subpopulations revealed characteristic alterations in infection dynamics following ASFV challenge (Figure 5c), with SusceptibleMac and RemodelMac exhibiting pronounced trajectory modifications, reflecting their enhanced viral permissiveness.

Trajectory analysis identified multiple discrete cellular states corresponding to distinct macrophage subpopulations and functional transitions (Figure 5f). State 3, characterized by elevated expression of DTX2, POLR3H, SLC16A10, CNTN3, LPAR1, AFAP1L1, and LTC4S, demonstrated significant enrichment in eicosanoid biosynthesis, fatty acid metabolism, monocarboxylic acid production, and transmembrane receptor protein serine/threonine kinase signaling pathways (Figure 5g). This state predominated in uninfected macrophages, suggesting functional specialization in lipid and eicosanoid metabolism during early infection stages. State 5, defined by marker genes including STAU2, ADORA2A, TMEM170B, I9R, A1CF, SLC24A4, and CEP83, showed enrichment in feeding behavior regulation, amelogenesis, embryonic implantation, and membrane repolarization pathways. This state was predominantly observed during advanced infection stages within SusceptibleMac and RemodelMac populations, indicating transition toward specialized cellular functions in response to viral challenge. State 6, marked by the expression of MAP4K4, THBS1, IL1R2, and S100A8, exhibited significant enrichment in apoptotic signaling pathways, particularly extrinsic apoptotic signaling regulation and negative proteolysis regulation. This state emerged during late infection stages, signifying activation of cell death mechanisms in response to viral stress. Notably, state 6 was also detected at reduced levels in uninfected RepairMac cells, suggesting potential involvement in cellular remodeling and tissue damage response independent of active viral infection.

Subpopulation-specific pseudotemporal analyses were performed for global infection processes (Figure S6), SusceptibleMac (Figure S7), RemodelMac (Figure S8), and RepairMac (Figure S9). These analyses consistently demonstrated that complement activation and immunoglobulin-mediated immune responses were exclusively associated with early infection stages, while nucleotide processing and monoatomic cation transport pathways clustered in late infection stages, establishing a temporal framework for functional transitions during ASFV progression.

### 3.6. Transcriptional Regulation and Cellular Crosstalk in Macrophage Subsets During ASFV Infection

**Figure 6:**
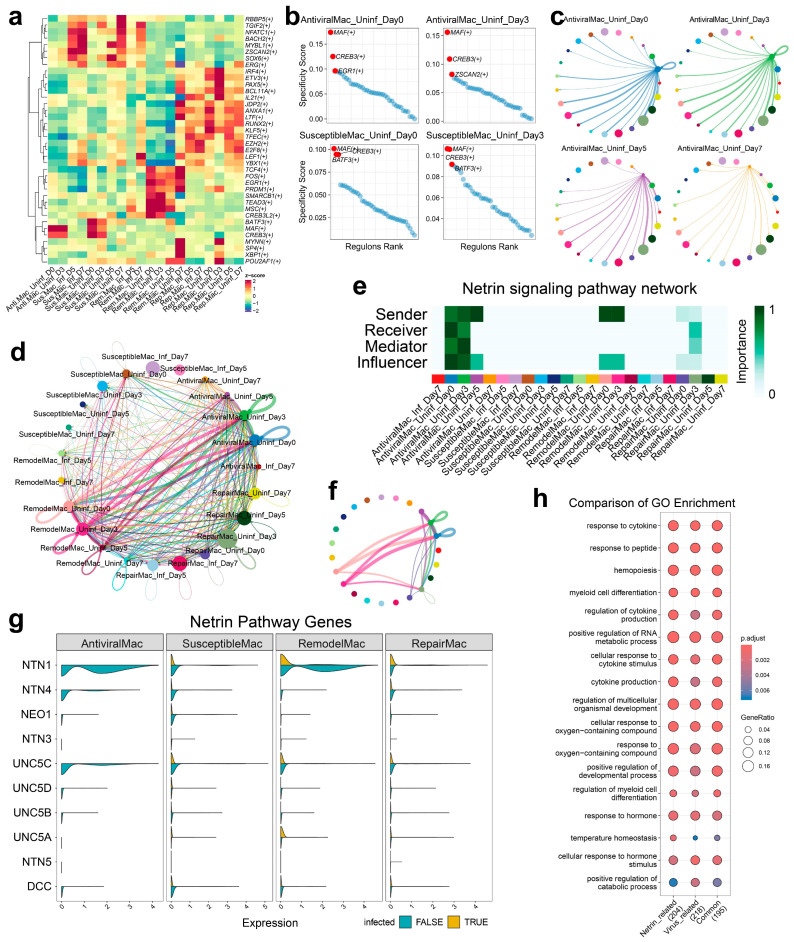
Transcriptional regulation and cellular crosstalk in macrophage subsets during ASFV infection. (**a**) Heatmap showing transcription factors (TFs) significantly enriched in infected versus uninfected cells across macrophage subsets and time points. (**b**) Ranked regulon activity of key TFs in AntiviralMac and SusceptibleMac during early infection. (**c**) Cell–cell communication network specific to AntiviralMac, highlighting ligand–receptor interactions. (**d**) Global communication patterns among all macrophage subsets, with edge weights indicating interaction strength. (**e**) Heatmap of Netrin signaling pathway interactions between macrophage subsets. (**f**) Circos plot visualizing Netrin-mediated crosstalk between macrophage subsets. (**g**) Expression profiles of Netrin pathway genes in infected versus uninfected cells across subtypes. (**h**) Comparative GO term enrichment analysis of Netrin signaling genes and ASFV infection-associated genes, revealing shared and distinct biological processes.

Analysis of transcriptional regulatory networks revealed distinct patterns of transcription factor (TF) activity across macrophage subpopulations during ASFV infection (Figure 6a). Hierarchical clustering demonstrated that TF profiles segregated primarily by cellular subtype rather than infection status. BATF3, MAF, and CREB3 exhibited significant enrichment in both AntiviralMac and SusceptibleMac subsets, suggesting their critical roles in modulating cellular responses to ASFV challenge. Ranked regulon activity analysis of key TFs during early infection further confirmed the predominant contributions of BATF3, MAF, and CREB3 within AntiviralMac and SusceptibleMac populations (Figure 6b and Figure S5c). These transcription factors are established regulators of myeloid cell immune responses, indicating their involvement in orchestrating host defense against viral invasion.

Intercellular communication network analysis revealed substantial alterations in cellular crosstalk during infection progression. Pseudotemporal trajectory analysis demonstrated marked reduction in intercellular signaling within AntiviralMac cells from Day 0 to Day 7 post-infection (Figure 6c). This progressive decline in cellular communication may reflect targeted disruption of macrophage signaling capabilities during infection, suggesting a potential viral strategy to compromise coordinated immune responses. Global communication pattern analysis revealed hierarchical differences in interaction strength among macrophage subsets, with AntiviralMac exhibiting maximal intercellular communication, followed sequentially by RemodelMac, RepairMac, and SusceptibleMac (Figure 6d). Combining with observed communication dynamics in AntiviralMac cells, the hierarchical relationship suggested that maintenance of robust intercellular signaling networks may constitute a critical determinant of resistance to ASFV infection.

Pathway-specific analysis identified Netrin signaling as significantly modulated during infection. Heatmap and circos plot analyses revealed enhanced Netrin-mediated interactions in AntiviralMac and RemodelMac populations during early infection stages (Figure 6e,f). Notably, Netrin pathway gene expression was substantially downregulated in these same cellular subsets following ASFV infection (Figure 6g), particularly for critical signaling components including NTN4, DCC, and NEO1. The Netrin signaling pathway regulated diverse cellular processes including migration, adhesion and survival. While research on Netrin-1 in splenic biology remains limited, existing studies suggest that it may influence macrophage polarization and immune modulation in this tissue [27]. NTN4 functions as a ligand for receptors including DCC and NEO1, which primarily mediate axon guidance and tissue morphogenesis during development but have recently been implicated in immune cell function. The significant downregulation of these genes in AntiviralMac and RemodelMac populations post-infection suggested targeted disruption of Netrin signaling as a potential viral mechanism for compromising macrophage functionality. Comparative gene ontology analysis of Netrin-related genes (Spearman ρ > 0.4, 204), virus-associated genes (Spearman ρ > 0.4, 218) with host genes, and genes common to both processes (195) revealed remarkable functional convergence (Figure 6h and Figures S14 and S15, File S1). These gene sets demonstrated highly similar enrichment profiles, particularly in biological processes related to hematopoiesis and myeloid cell differentiation (Figure S10). This substantial functional overlap suggested that ASFV may specifically target pathways critical for myeloid cell development and functionality, with Netrin signaling representing a key component of this interaction.

## 4. Discussion

This study presented a comprehensive single-cell transcriptomic analysis of the dynamic cellular landscape in the spleen during ASFV infection, revealing critical insights into viral tropism, host immune responses, and the functional heterogeneity of macrophage subpopulations. Our findings highlighted the pivotal role of macrophages as the primary cellular reservoir for ASFV replication and uncover distinct macrophage subsets with varying susceptibility to infection, transcriptional reprogramming, and functional specialization. Importantly, the observed transcriptional changes in infected macrophages may reflect not only host immune responses but also direct manipulation by ASFV’s cytoplasmic replication cycle, particularly through the formation of perinuclear viral factories that hijack host organelles and metabolic pathways [3]. These results provided a detailed cellular and molecular framework for understanding ASFV pathogenesis and host–pathogen interactions.

Consistent with previous reports, our data confirmed that ASFV exhibits a strong tropism for macrophages [9,28,29], with viral transcripts predominantly localized to these cells, particularly at later stages of infection (Days 5 and 7). The progressive accumulation of viral load in macrophages, coupled with the selective expression of key ASFV genes, including MGF-100-2L, K205R [24], and CP204L [30], underscores their role of virulence of the virus and immune evasion as the major viral replication niche. The gradient decline of viral replication-associated genes (E310R [31] and M1249L [32]) across macrophage subsets—from highly expressed in SusceptibleMac to diminished in RemodelMac and nearly absent in RepairMac—suggests a direct link between ASFV’s intracellular replication cycle and macrophage functional polarization. The top 10 ASFV genes identified in our study (MGF-300-4L, E296R, E165R, MGF-110-2L, CP312R, EP152R, MGF-110-5L, CP204L, K205R, and MGF-100-2L) play critical roles in viral replication, immune evasion, and structural assembly. Key findings include the following: (1) Early genes (e.g., CP204L [33], EP152R [34]) facilitate viral entry and initial infection; (2) immune modulators (e.g., MGF-100-2L, MGF-110-5L [35]) suppress host interferon responses, explaining macrophage-specific tropism; and (3) Late-stage genes (e.g., K205R [24], CP312R [36]) drive virion production. These results align with our observation of progressive infection in macrophages (Days 5–7) and highlight potential therapeutic targets (e.g., E165R for replication inhibition). Notably, the near-exclusive colocalization of high viral loads with the SusceptibleMac subset, which expanded significantly over time, suggested that this subpopulation is particularly linked to ASFV’s reliance on host energy and biosynthetic resources for viral factory assembly. This finding not only aligned with prior studies demonstrating macrophage susceptibility to ASFV, but also extended these observations by identifying specific subpopulations that drive viral propagation. The concurrent depletion of AntiviralMac (ASFV−, interferon-activated, Figure S13) cells, which were likely involved in early host defense, further suggested that ASFV actively suppresses protective immune responses to establish infection [37,38].

While our subclustering revealed four novel macrophage subsets (SusceptibleMac, RepairMac, RemodelMac, AntiviralMac), these populations do not strictly align with traditional M1/M2 classifications (Figures S11 and S12). Notably, AntiviralMac displayed paradoxical co-expression of PPARG (typically associated with M2-like polarization [39]) alongside STAT1 (M1-like pro-inflammatory markers [40]), suggesting an antiviral but metabolically dysregulated state. RepairMac simultaneously upregulated canonical M1 markers (CD86, CXCL10) and M2-associated factors (TGFB1, IL10), indicative of a hybrid inflammation-resolution phenotype. SusceptibleMac showed no clear polarization bias but uniquely expressed interferon-stimulated genes (ISG15, MX1), suggesting a metabolically dysregulated state. This complex rewiring mirrors observations in HIV-infected macrophages, where viral interference creates “polarization-less” states that evade immune detection [41].

Our analysis revealed extensive transcriptional reprogramming in infected macrophages, characterized by the upregulation of interferon-stimulated genes (MX1, ISG15, IRF7, CXCL10) and inflammatory mediators (TNF, IL1B, CCL2), indicative of a robust type I interferon response [42,43]. However, the strong positive correlation between antiviral genes and viral burden suggested that ASFV may exploit these pathways to facilitate replication, as previously proposed for other viral infections [44,45,46]. Conversely, the downregulation of genes involved in antigen presentation (CD80, CD86, HLA-DRB1) and T cell activation (CD4, CD8A, CCL5) points to potential immune evasion mechanisms [1,47], impairing adaptive immune recognition and response. The divergent correlation patterns of CD86 (negative in macrophages but positive in other cells) further highlighted macrophage-specific regulatory disruptions during infection.

Subclustering analysis uncovered four functionally distinct macrophage subsets with unique transcriptional signatures and infection dynamics. The enrichment of oxidative phosphorylation and ATP synthesis pathways in SusceptibleMac suggests that ASFV may hijack cellular metabolic processes to meet its energy demands, a strategy observed in other viral infections [48]. In contrast, RepairMac cells, associated with tissue repair and inflammation resolution, exhibited stable transcription factor networks despite infection, implying a role in maintaining tissue homeostasis during pathogenesis. The near-complete loss of AntiviralMac populations by Day 5 post-infection raised intriguing questions about whether ASFV directly targets these cells for elimination or induces their transition into other states [49], such as SusceptibleMac or RemodelMac. The latter subset, which emerged as a secondary viral reservoir with distinct tropism for MGF genes, may represent a transitional state influenced by viral-induced remodeling of host pathways.

Pseudotemporal trajectory analysis revealed a continuum of macrophage states, with uninfected cells predominantly associated with lipid metabolism (State 3) and infected cells transitioning toward pro-viral (State 5) or apoptotic (State 6) fates. The late emergence of State 6, marked by apoptotic signaling genes (THBS1, IL1R2, S100A8), suggested that ASFV may eventually trigger cell death in heavily infected macrophages, potentially contributing to the cytopathic effects observed in advanced infection [50]. The shift in viral gene expression from SusceptibleMac to RemodelMac between Days 5 and 7 further implied dynamic changes in cellular permissiveness, possibly driven by viral modulation of host factors or immune pressure. Another striking finding was the progressive communicative isolation of SusceptibleMac, which lost detectable intercellular signaling by Day 7. This reflected viral disruption of immune coordination networks [1,51,52], limiting the ability of infected macrophages to recruit or alert other immune cells.

Netrins are a family of secreted proteins known to function primarily as axon guidance molecules in the nervous system [53], but they also play important roles in tissue morphogenesis [54], immune cell function [16], and inflammation regulation [15] outside the nervous system. Notably, Netrin-1 has been shown to influence macrophage migration and survival through its receptors [14,55], suggesting a potential immunomodulatory function. While reports on Netrin signaling during viral infections are limited, a previous study demonstrated mutual amplification between Netrin-1 and hepatitis C virus via epidermal growth factor receptor pathways [56], indicating that Netrins can be co-opted during viral pathogenesis. Given the overlap between Netrin-related genes and those involved in myeloid differentiation, this disruption could impair the generation or function of protective macrophage subsets, favoring viral persistence. The downregulation of Netrin signaling components (NTN4, DCC, NEO1) in AntiviralMac and RemodelMac subsets further suggested that ASFV targets pathways critical for macrophage migration, adhesion, and survival. This finding opens new avenues for understanding how ASFV manipulates host cell signaling networks to facilitate viral replication and evasion of immune clearance.

Our study identified SusceptibleMac and RemodelMac as key reservoirs for ASFV replication, with E165R and E296R emerging as central nodes in viral gene networks. The critical role of E165R, validated by prior studies, highlights its potential as a therapeutic target. Additionally, the depletion of AntiviralMac and the metabolic reprogramming of SusceptibleMac suggested that interventions preserving antiviral macrophage subsets or modulating cellular metabolism could mitigate infection. The disruption of Netrin signaling further unveiled a novel mechanism of ASFV immune evasion, offering new avenues for host-directed therapies. While this study provided high-resolution insights into spleen immune responses, further work was needed to determine whether these findings extend to other tissues or ASFV strains. Functional validation of macrophage subsets and their roles in viral dissemination, as well as mechanistic studies targeting E165R or Netrin signaling, will be essential to translate these findings into practical interventions.

While our study provides high-resolution insights into spleen immune responses, we acknowledge that the scRNA-seq approach cannot fully resolve spatial aspects of viral replication (e.g., viral factory proximity to specific organelles). Future studies integrating spatial transcriptomics or multiplexed imaging will be critical to dissect how viral factory formation directly shapes local transcriptional niches in infected macrophages. While this study provided high-resolution insights into spleen immune responses, further work was needed to determine whether these findings extend to other tissues or ASFV strains. Functional validation of macrophage subsets and their roles in viral dissemination, as well as mechanistic studies targeting E165R or Netrin signaling, combined with direct visualization of viral factory–host interactions, will be essential to translate these findings into practical interventions.

## 5. Conclusions

In summary, our single-cell transcriptomic analysis delineates the dynamic remodeling of the spleen immune microenvironment during ASFV infection, emphasizing the central role of macrophage heterogeneity in viral pathogenesis. By identifying susceptible subsets, transcriptional networks, and disrupted signaling pathways, this work advances our understanding of ASFV-host interactions and provides a foundation for developing targeted strategies to combat this devastating pathogen.

## Data Availability

The original data presented in the study are openly available in the GEO database with accession numbers PRJNA879060.

## Supplementary Materials

The following supporting information can be downloaded at: https://www.mdpi.com/article/10.3390/biology14070882/s1, Supplementary Figure S1. Temporal analysis of cellular composition of every cell type in spleen during ASFV infection. (a) Cellular composition changes in every sample of every cell type. (b) Infection thresholds determined separately for each stage in all cell types. (c) Infection thresholds determined separately for each stage in macrophages. Supplementary Figure S2. Viral dynamics of ASFV expression across different time points post-challenge. (a) ASFV infection rate and mean viral load in every cell type. (b) Viral load across every time points. (c) Correlation analysis of total viral load of viral genes. Supplementary Figure S3. Correlation analysis between host gene module expression and viral load. (a) Correlation analysis between host gene module expression and viral load in infected cells. (b) Core gene expressing pattern of host gene modules. Supplementary Figure S4. Viral gene expressing patterns along ASFV infection. Supplementary Figure S5. Gene expressing patterns in infected sub macrophages. (a,b) Marker genes and enriched pathways for different infected sub macrophages in day5 (a), day7 (b). (c,d) Regulons rank of transcription factors in every sub macrophage before infecting (c), and after infecting (d). (e) Core regulon effect of CREB3 in infected macrophages. (f) Core regulon effect of XBP1 in uninfected macrophages. Supplementary Figure S6. Pseudotemporal Analysis of infected Macrpphages During ASFV Infection. (a) Overall trajectory of infected Macrpphages in pseudotime. (b) Detailed trajectory plots for each macrophage subtype before and after ASFV infection. (c) Trajectory plots for each macrophage subtype in day5 and day7. (d) Distribution of different cell states across the infected Macrophages pseudo temporal trajectory. (g) Marker genes and enriched pathways for different cell states identified through pseudotemporal analysis. Supplementary Figure S7. Pseudotemporal Analysis of SusceptibleMac During ASFV Infection. (a) Overall trajectory of SusceptibleMac in pseudotime. (b) Detailed trajectory plots for each SusceptibleMac subtype before and after ASFV infection. (c) Trajectory plots for SusceptibleMac subtype along ASFV infection. (d) Distribution of different cell states and Marker genes and enriched pathways for different cell states identified through pseudotemporal analysis. (e,f) The gene-normalized dynamics of selected genes along the pseudotime trajectories during infecting (e) and different stage (f). Each dot represents a single cell that is color-coded by timepoints. (g) Cellular communication changes of SusceptibleMac in every subtypes. Supplementary Figure S8. Pseudotemporal Analysis of RemodelMac During ASFV Infection. (a) Cellular communication changes of RemodelMac in every subtypes. (b) Overall trajectory of RemodelMac in pseudotime. (c) Detailed trajectory plots for each RemodelMac subtype before and after ASFV infection. (d) Trajectory plots for RemodelMac subtype before infecting. (e) Distribution of different cell states. (f) Marker genes and enriched pathways for different cell states identified through pseudotemporal analysis. Supplementary Figure S9. Pseudotemporal Analysis of RepairMac During ASFV Infection. (a) Cellular communication changes of RepairMac in every subtypes. (b) Overall trajectory of RepairMac in pseudotime. (c) Trajectory plots for RepairMac subtype before infecting. (d) Trajectory plots for RepairMac subtype along infecting. (e) Detailed trajectory plots for each RepairMac subtype before and after ASFV infection. (f) Distribution of different cell states. (g) Marker genes and enriched pathways for different cell states identified through pseudotemporal analysis. Supplementary Figure S10. GO enrichment analysis of virus and netrin-related genes. Supplementary Figure S11. Heatmapping of M1/M2 macrophages markers expressing in different macrophages subsets. Supplementary Figure S12. M1/M2 macrophages markers expressing in different macrophages subsets. Supplementary Figure S13. Interferon-related gene expression in different sub macrophages during infection. Supplementary Figure S14. GO Enrichment analysis of virus positively related host genes. Supplementary Figure S15. GO Enrichment analysis of Netrin pathway positively related host genes. File S1. Virus and Netrin related host genes.

## Abbreviations

The following abbreviations are used in this manuscript:

ASF	African swine fever
ASFV	African swine fever virus
PCA	Principal component analysis
TF	Transcription factors
UMAP	Uniform manifold approximation and projection
GO	Gene ontology
KEGG	Kyoto Encyclopedia of Genes and Genomes
DEGs	Differentially expressed genes
PPR	Pattern recognition receptor
