# Supplementary material for: Single-Cell Atlas of Spleen Remodeling Reveals Macrophage Subset-Driven ASFV Pathogenesis
Source: Biology (Basel). 2025 Jul 18;14(7):882. doi: 10.3390/biology14070882 (PMC12293015; doi:10.3390/biology14070882)

Supplementary Information

Single-cell atlas of splenic remodeling reveals macrophage subset-driven ASFV pathogenesis

This file includes Supplementary Figures S1 to S15.

Supplementary Figure S1. Temporal analysis of cellular composition of every cell type in spleen during ASFV infection

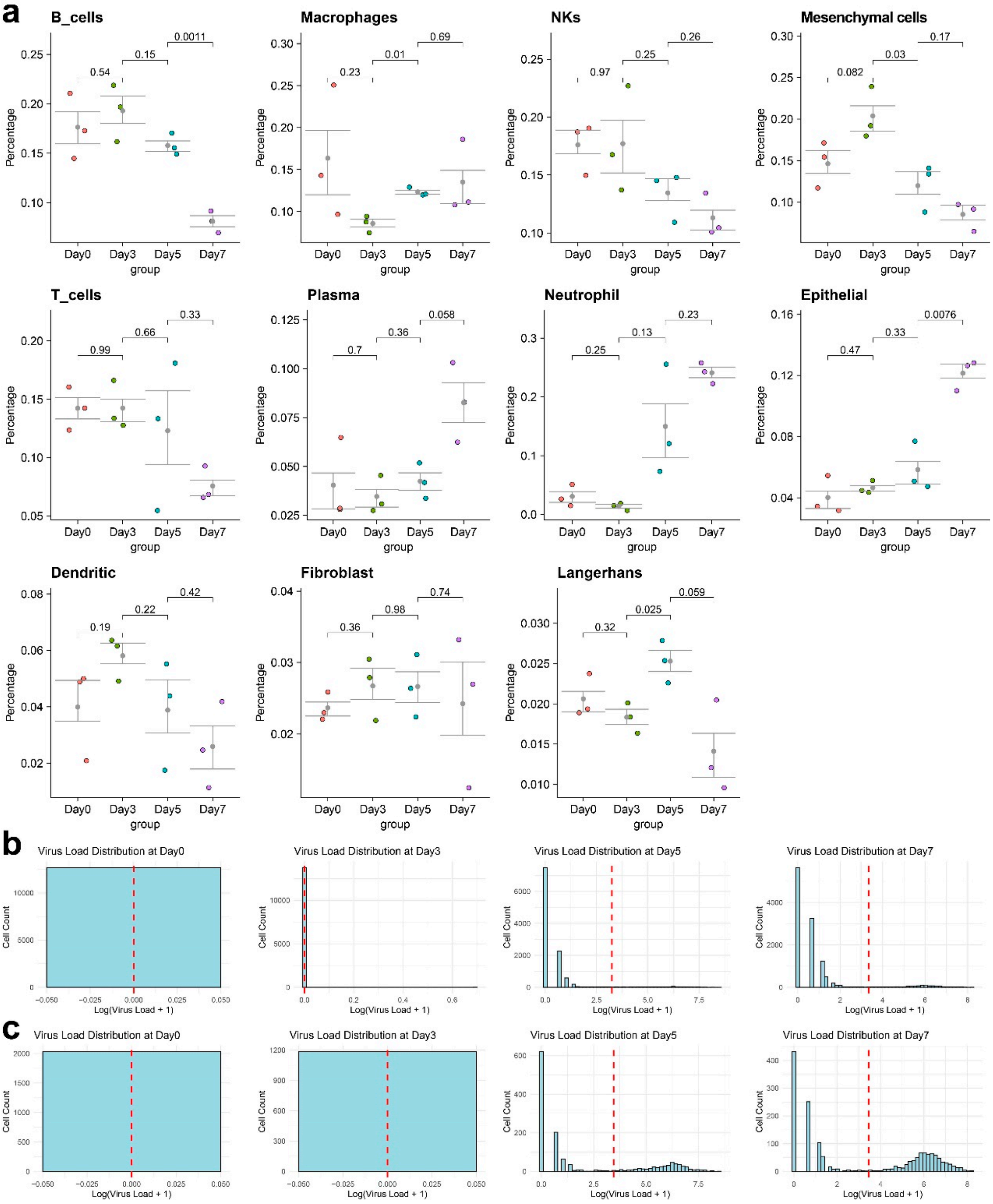

(a) Cellular composition changes in every sample of every cell type.

(b) Infection thresholds determined separately for each stage in all cell types.

(c) Infection thresholds determined separately for each stage in macrophages.

Supplementary Figure S2. Viral dynamics of ASFV expression across different time points post-challenge

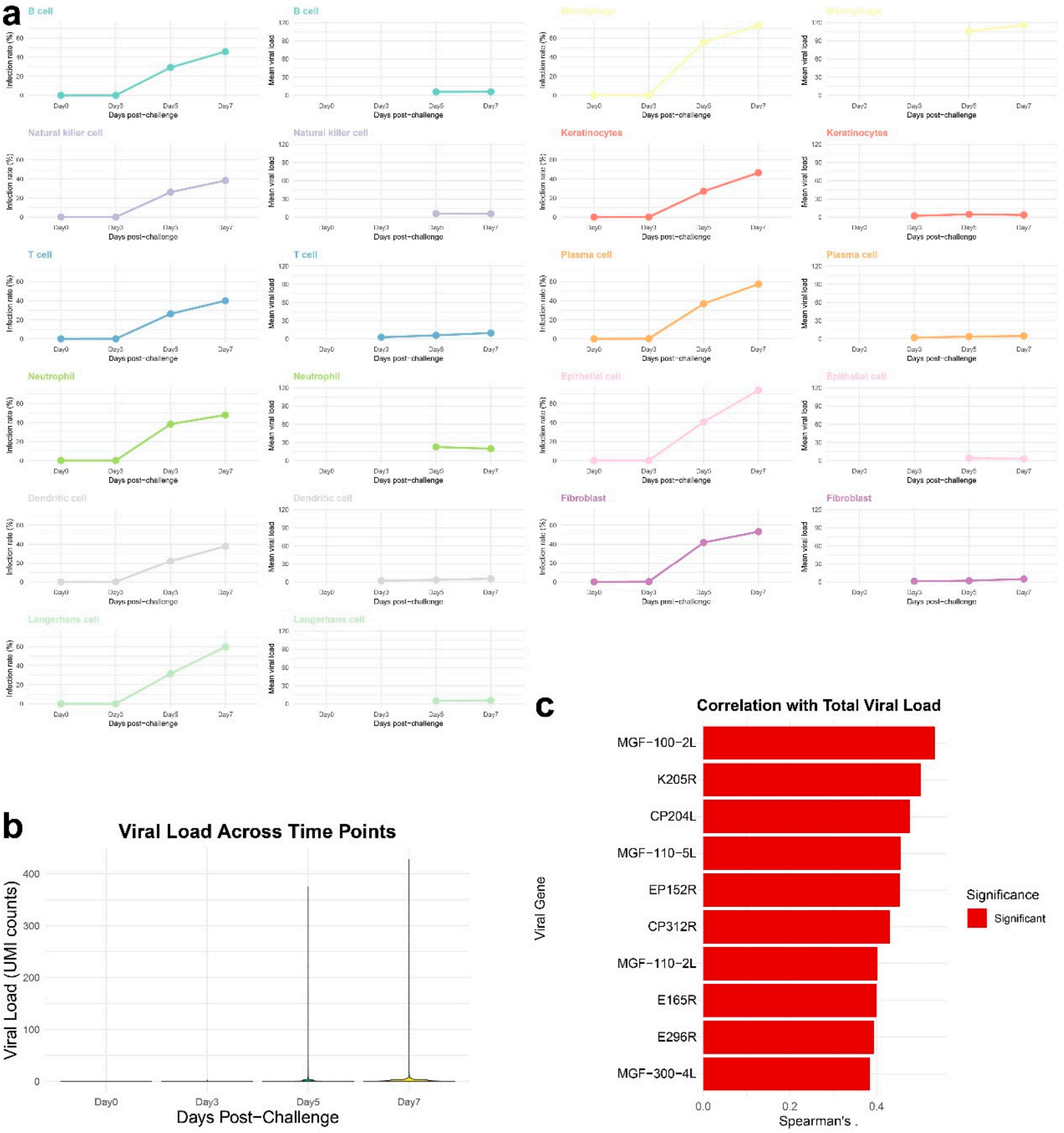

(a) ASFV infection rate and mean viral load in every cell type.

(b) Viral load across every time points.

(c) Correlation analysis of total viral load of viral genes.

Supplementary Figure S3. Correlation analysis between host gene module expression and viral load

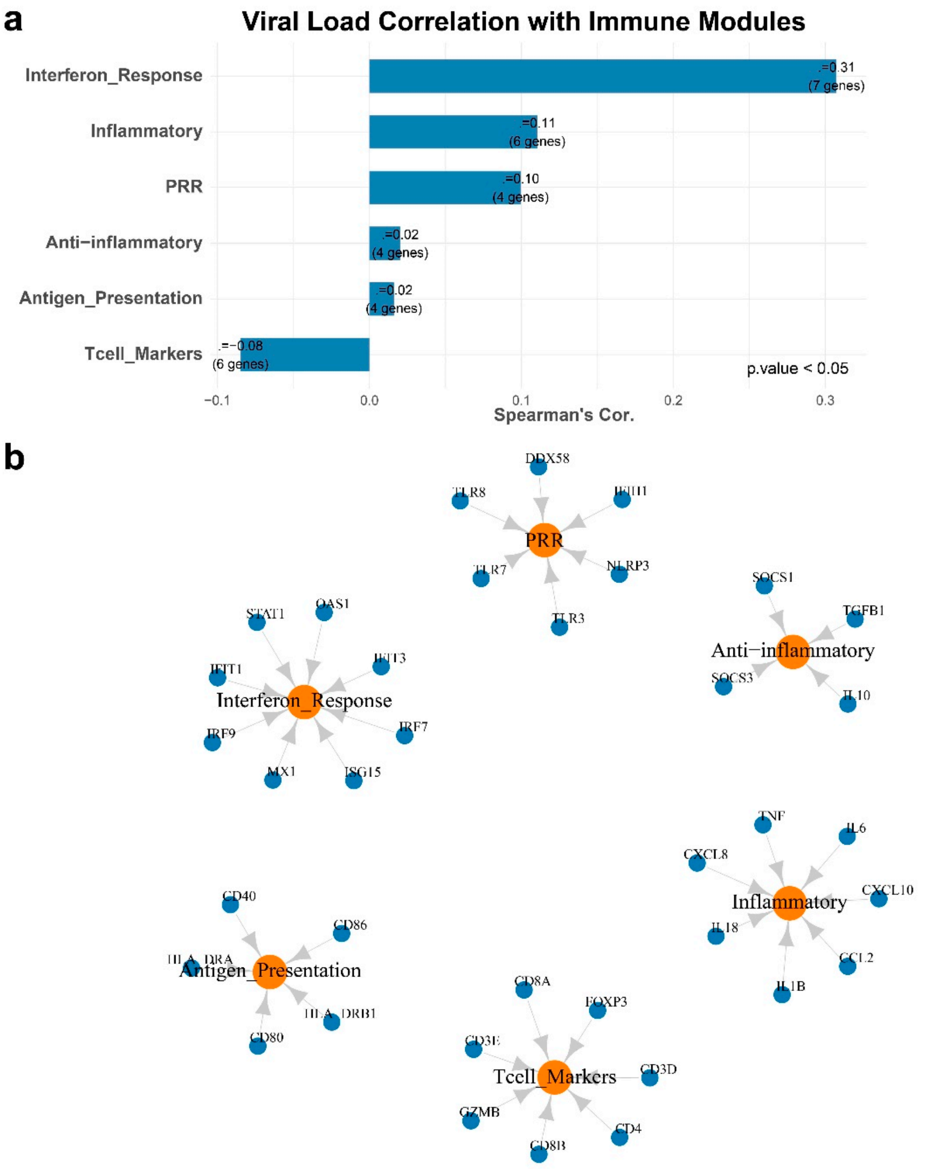

(a) Correlation analysis between host gene module expression and viral load in infected cells.

(b) Core gene expressing pattern of host gene modules.

Supplementary Figure S4. Viral gene expressing patterns along ASFV infection

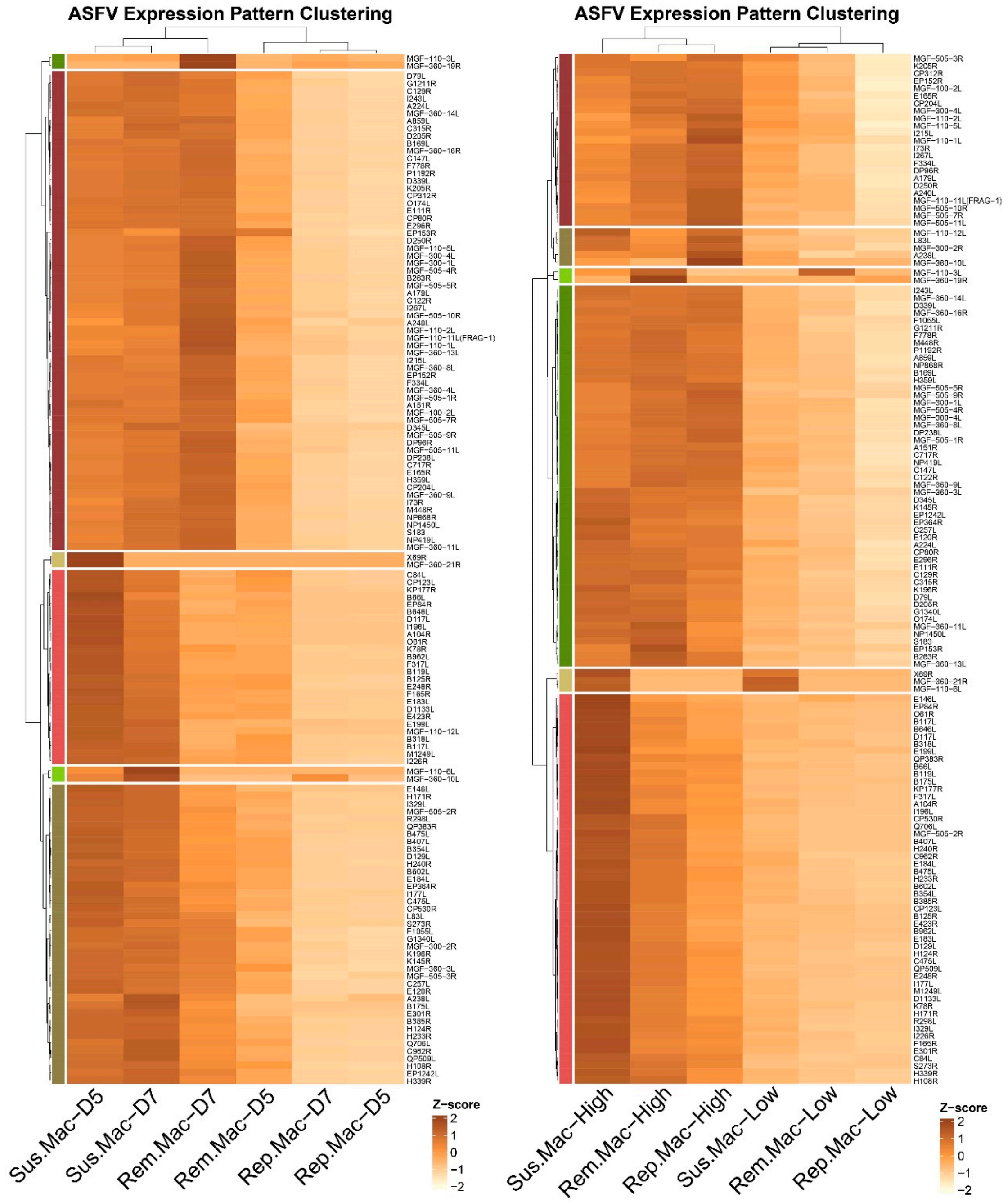

Supplementary Figure S5. Gene expressing patterns in infected sub macrophages

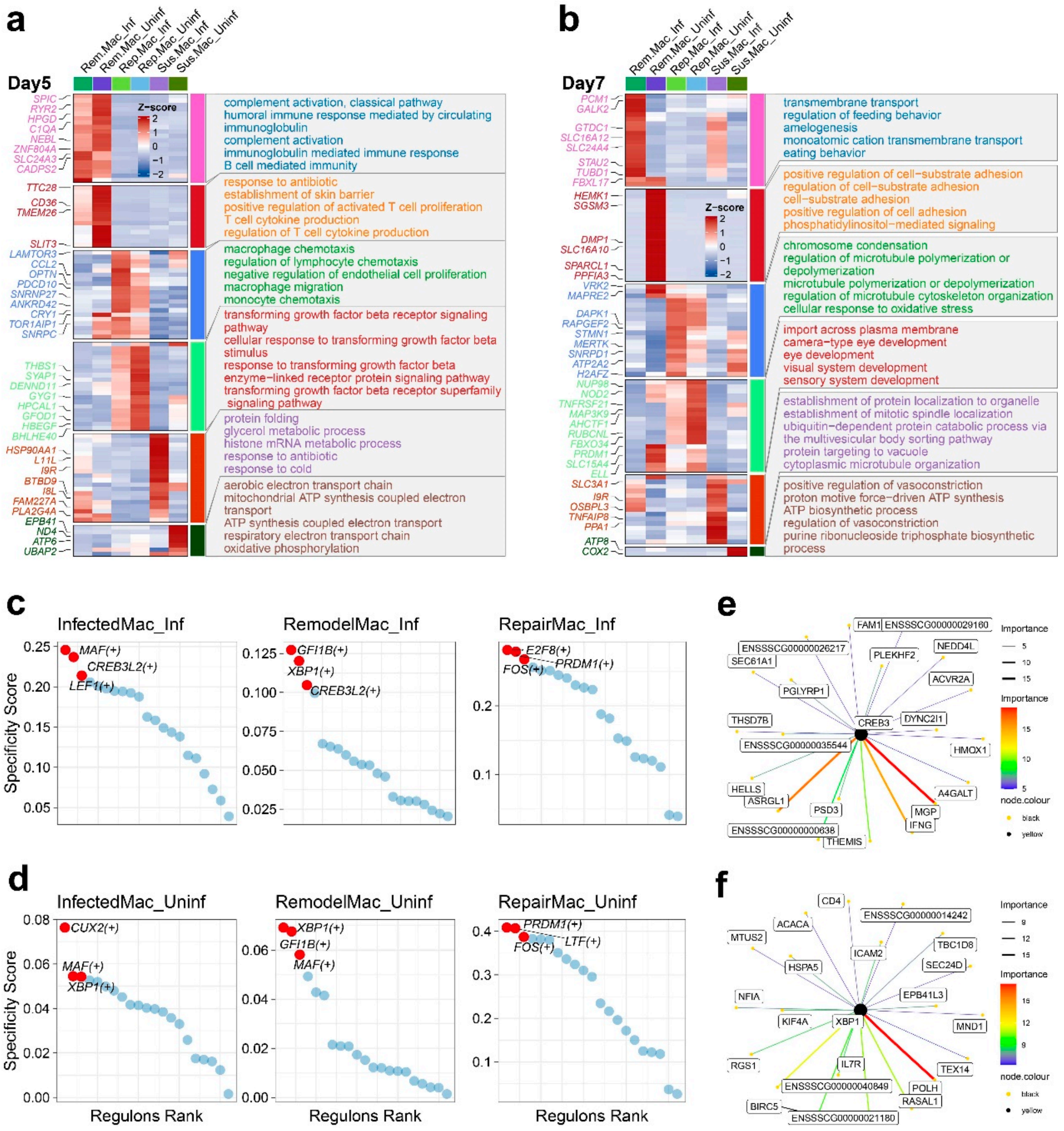

(a-b) Marker genes and enriched pathways for different infected sub macrophages in day5 (a), day7 (b).

(c-d) Regulons rank of transcription factors in every sub macrophage before infecting (c), and after infecting (d).

(e) Core regulon effect of CREB3 in infected macrophages.

(f) Core regulon effect of XBP1 in uninfected macrophages.

Supplementary Figure S6. Pseudotemporal Analysis of infected Macrpphages During ASFV Infection

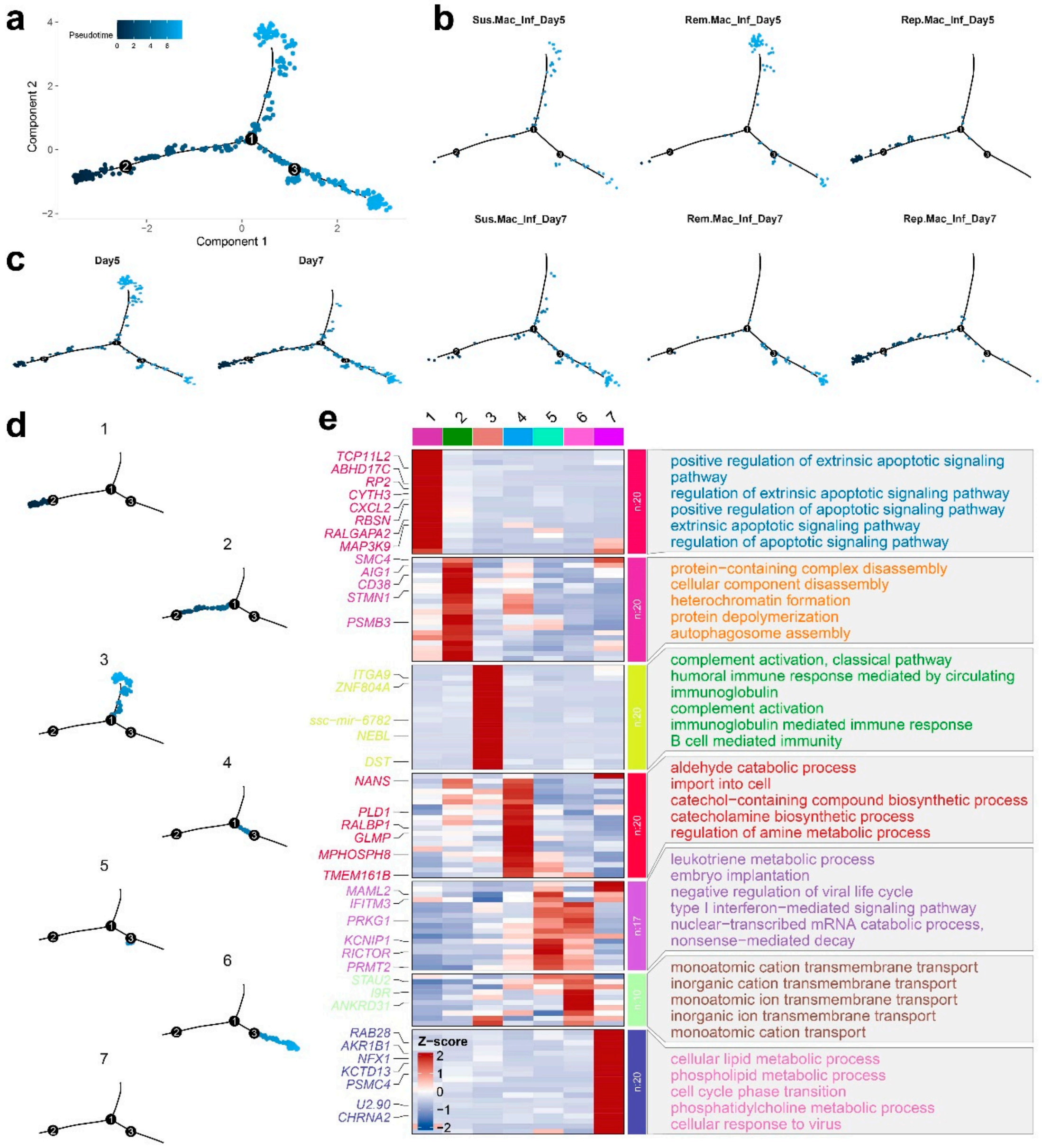

(a) Overall trajectory of infected Macrpphages in pseudotime.

(b) Detailed trajectory plots for each macrophage subtype before and after ASFV infection.

(c) Trajectory plots for each macrophage subtype in day5 and day7.

(d) Distribution of different cell states across the infected Macrophages pseudo temporal trajectory.

(g) Marker genes and enriched pathways for different cell states identified through pseudotemporal analysis.

Supplementary Figure S7. Pseudotemporal Analysis of SusceptibleMac During ASFV Infection

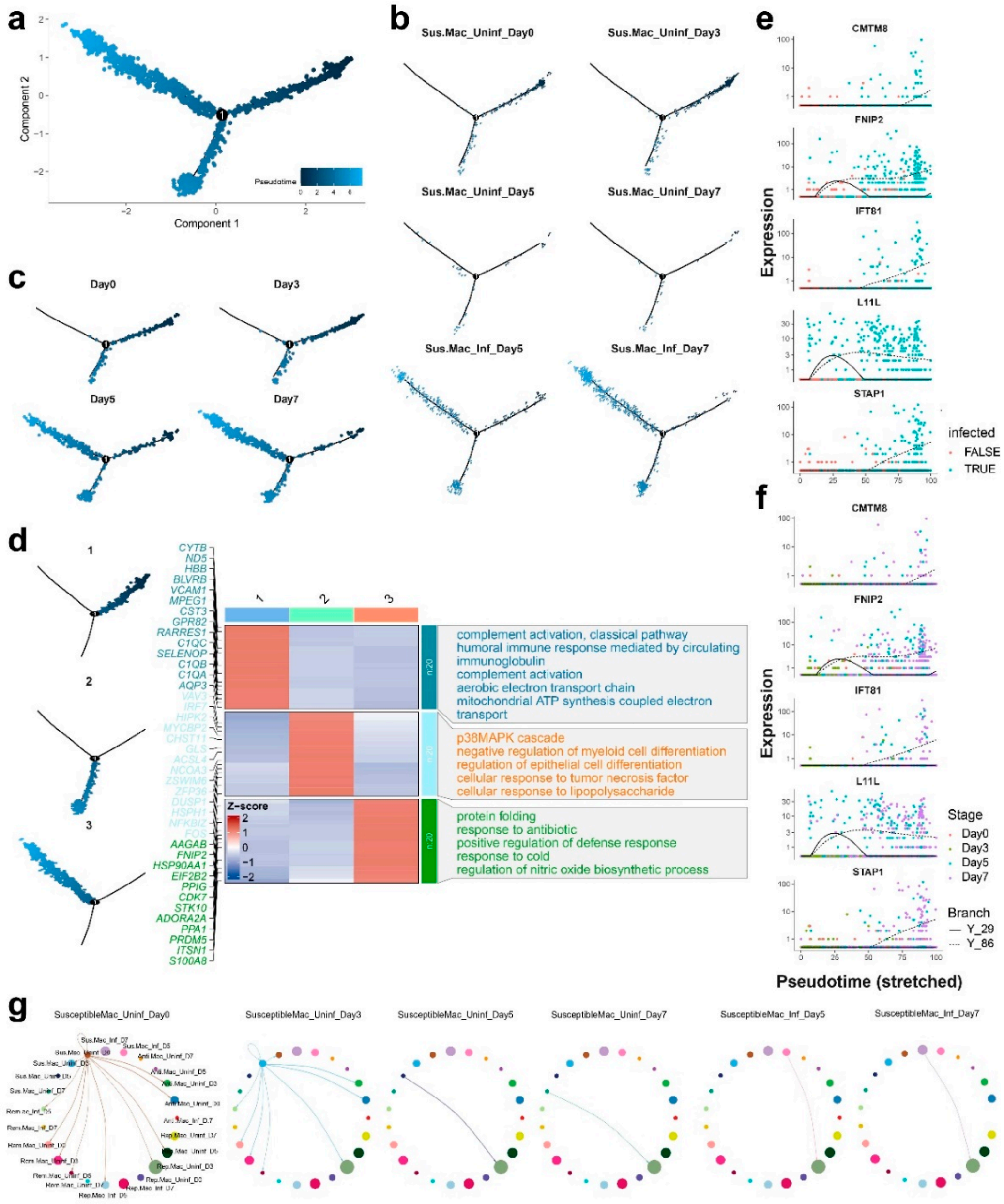

(a) Overall trajectory of SusceptibleMac in pseudotime.

(b) Detailed trajectory plots for each SusceptibleMac subtype before and after ASFV infection.

(c) Trajectory plots for SusceptibleMac subtype along ASFV infection.

(d) Distribution of different cell states and Marker genes and enriched pathways for different cell states identified through pseudotemporal analysis.

(e-f) The gene-normalized dynamics of selected genes along the pseudotime trajectories during infecting (e) and different stage (f). Each dot represents a single cell that is color-coded by timepoints.

(g) Cellular communication changes of SusceptibleMac in every subtypes.

Supplementary Figure S8. Pseudotemporal Analysis of RemodelMac During ASFV Infection

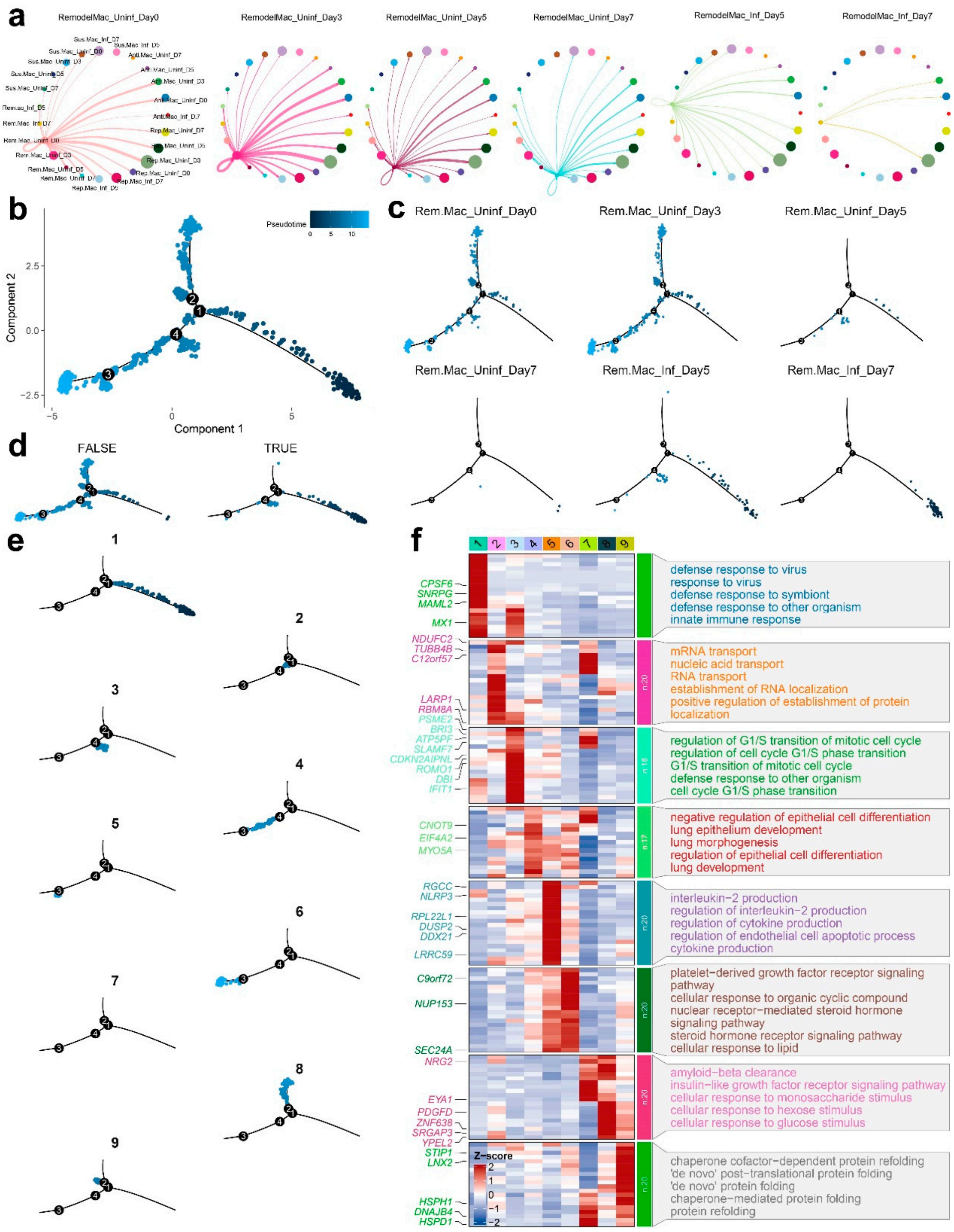

(a) Cellular communication changes of RemodelMac in every subtypes.

(b) Overall trajectory of RemodelMac in pseudotime.

(c) Detailed trajectory plots for each RemodelMac subtype before and after ASFV infection.

(d) Trajectory plots for RemodelMac subtype before infecting.

(e) Distribution of different cell states.

(f) Marker genes and enriched pathways for different cell states identified through pseudotemporal analysis.

Supplementary Figure S9. Pseudotemporal Analysis of RepairMac During ASFV Infection

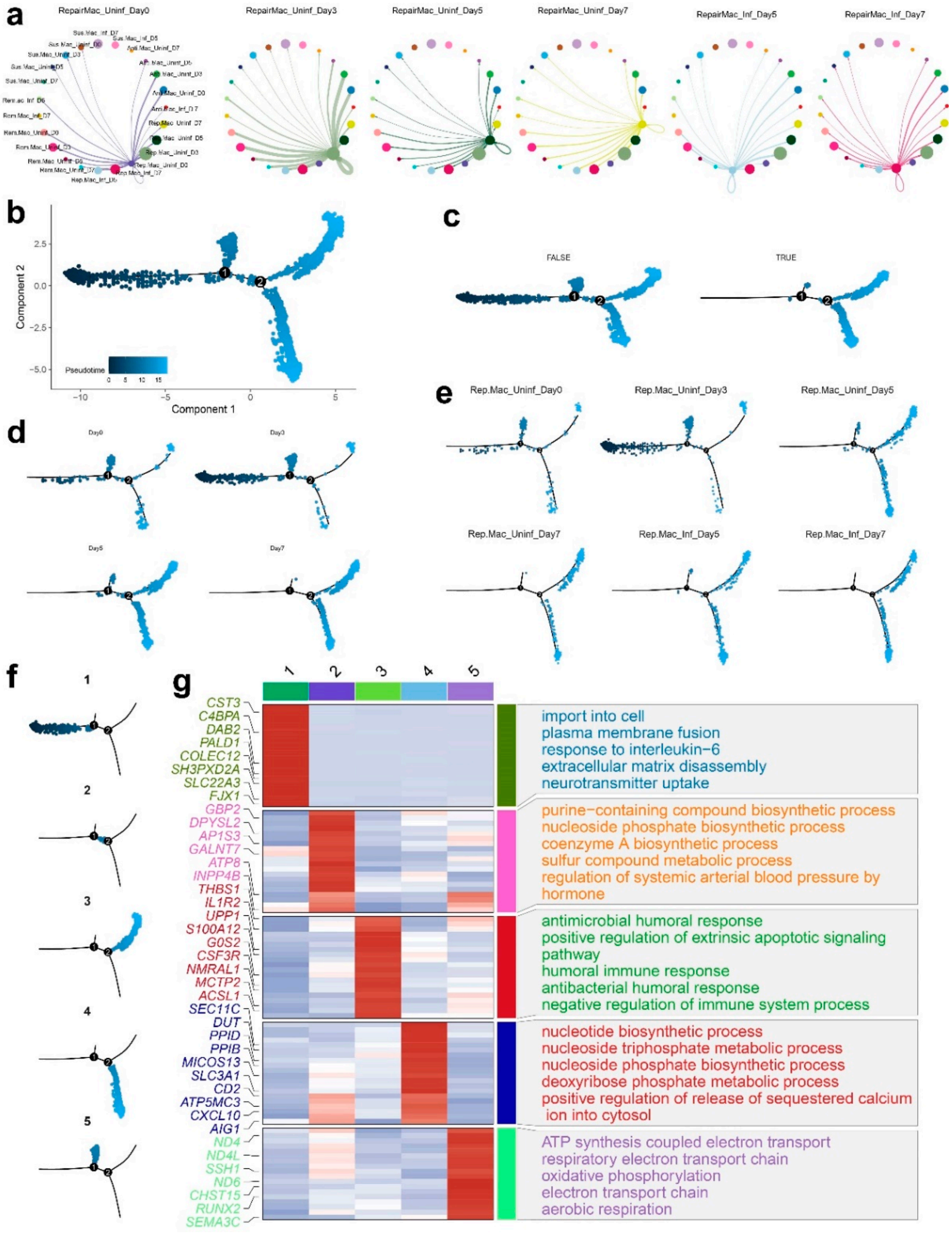

(a) Cellular communication changes of RepairMac in every subtypes.

(b) Overall trajectory of RepairMac in pseudotime.

(c) Trajectory plots for RepairMac subtype before infecting.

(d) Trajectory plots for RepairMac subtype along infecting.

(e) Detailed trajectory plots for each RepairMac subtype before and after ASFV infection.

(f) Distribution of different cell states.

(g) Marker genes and enriched pathways for different cell states identified through pseudotemporal analysis.

Supplementary Figure S10. GO enrichment analysis of virus and netrin-related genes

a

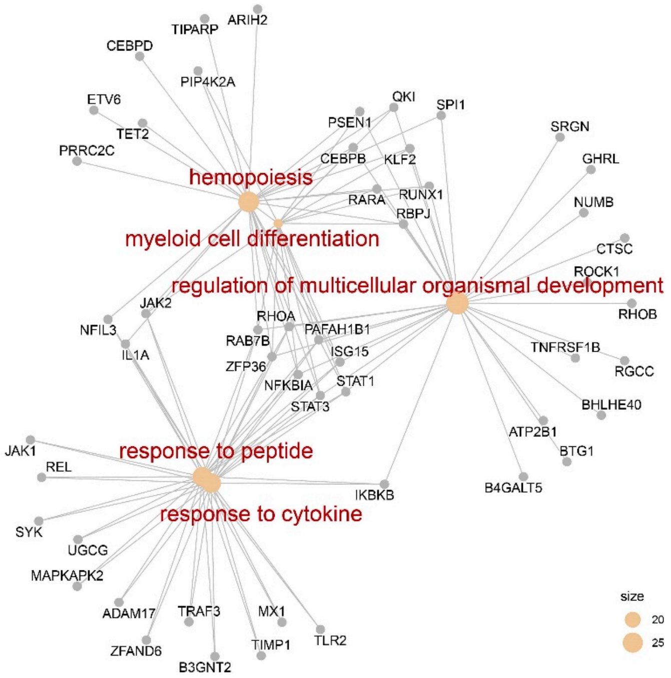

GO_enrichment of Virus_related genes

b

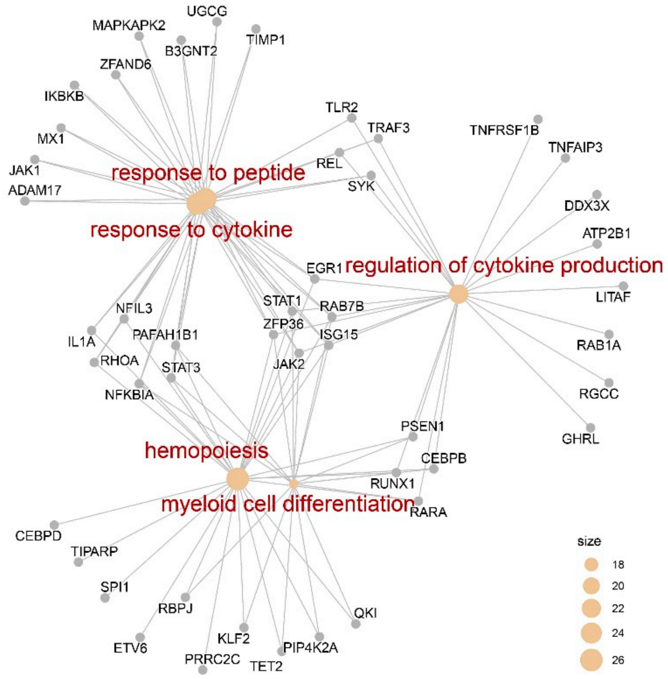

GO_enrichment of Netrin_related genes

Supplementary Figure S11. Heatmapping of M1/M2 macrophages markers expressing in different macrophages subsets.

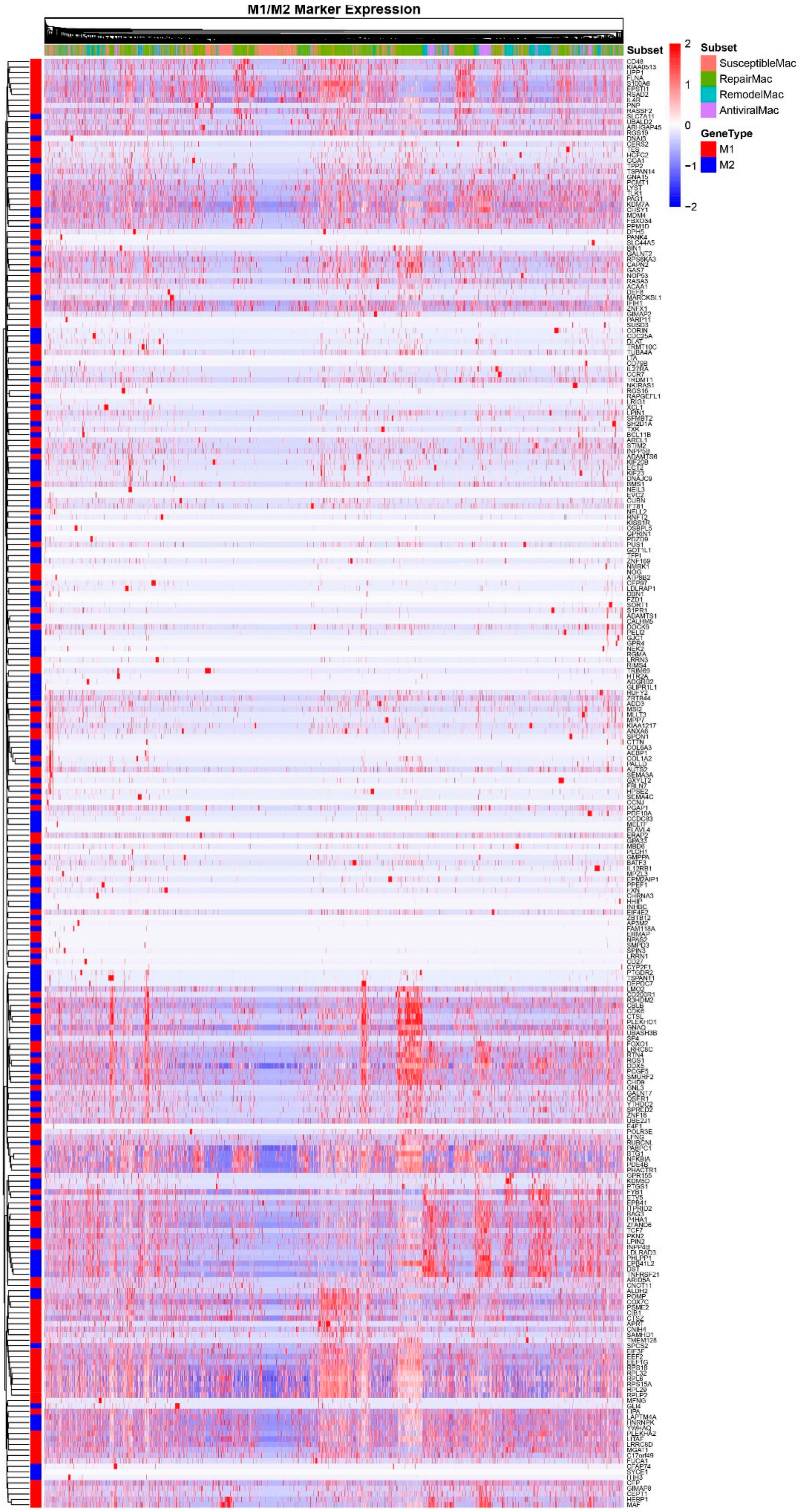

Supplementary Figure S12. M1/M2 macrophages markers expressing in different macrophages subsets.

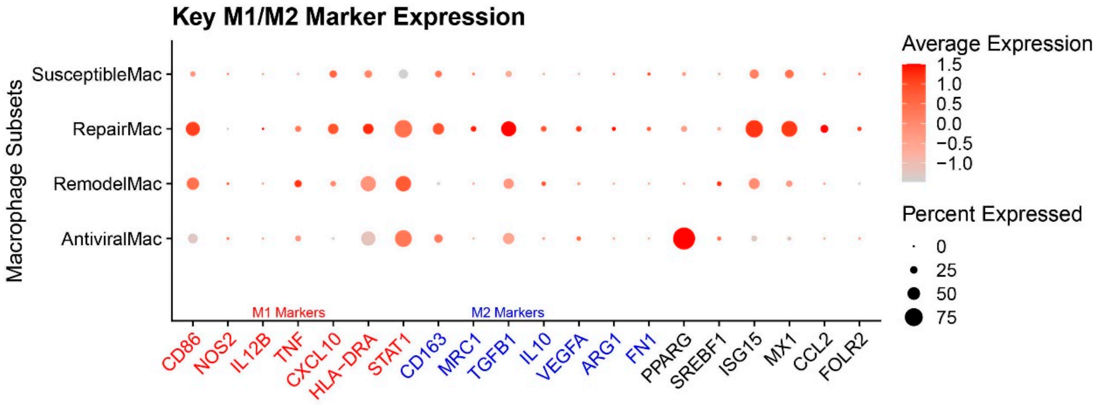

Supplementary Figure S13. Interferon-related gene expression in different sub macrophages during infection.

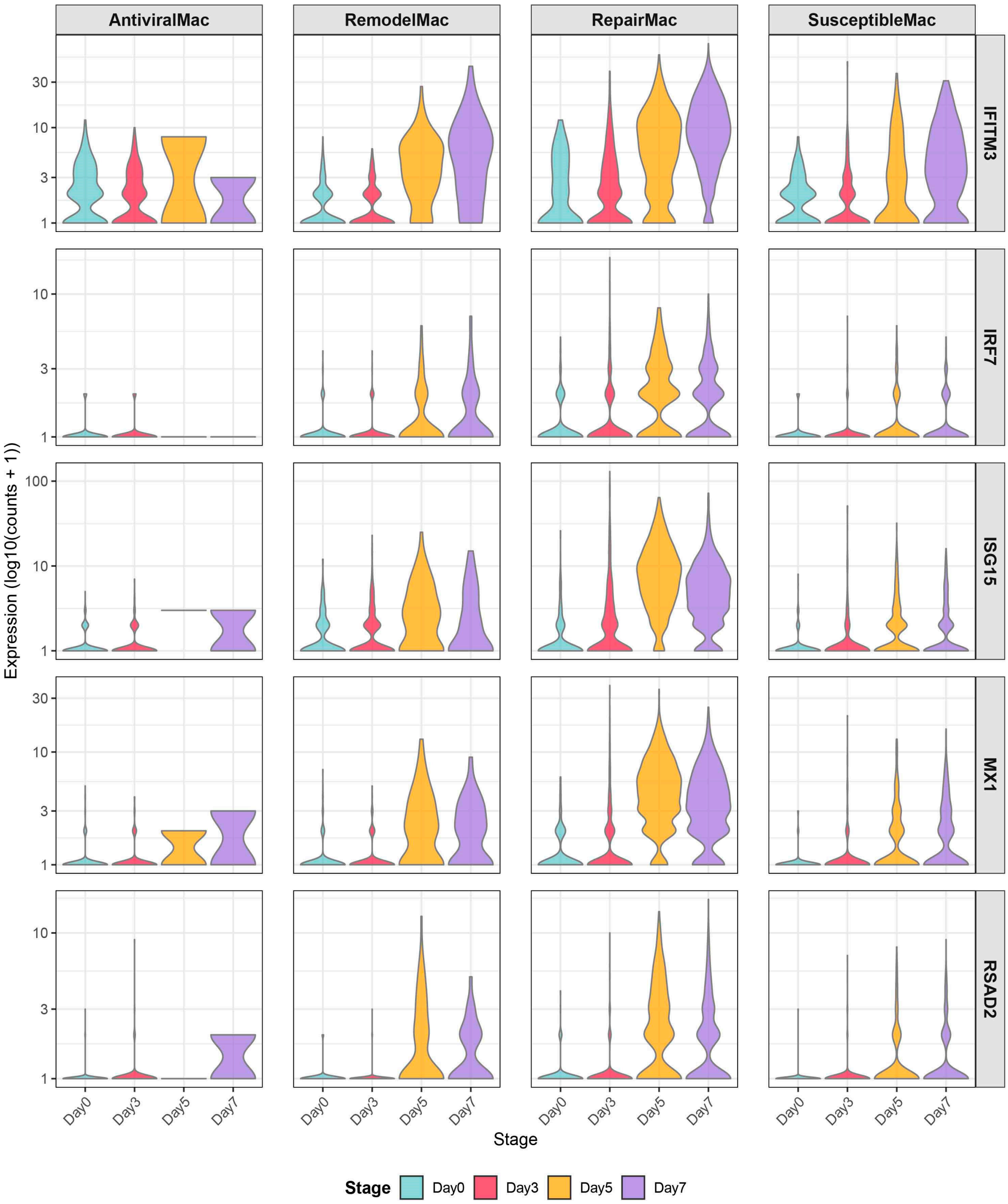

Supplementary Figure S14. GO Enrichment analysis of virus positively related host genes

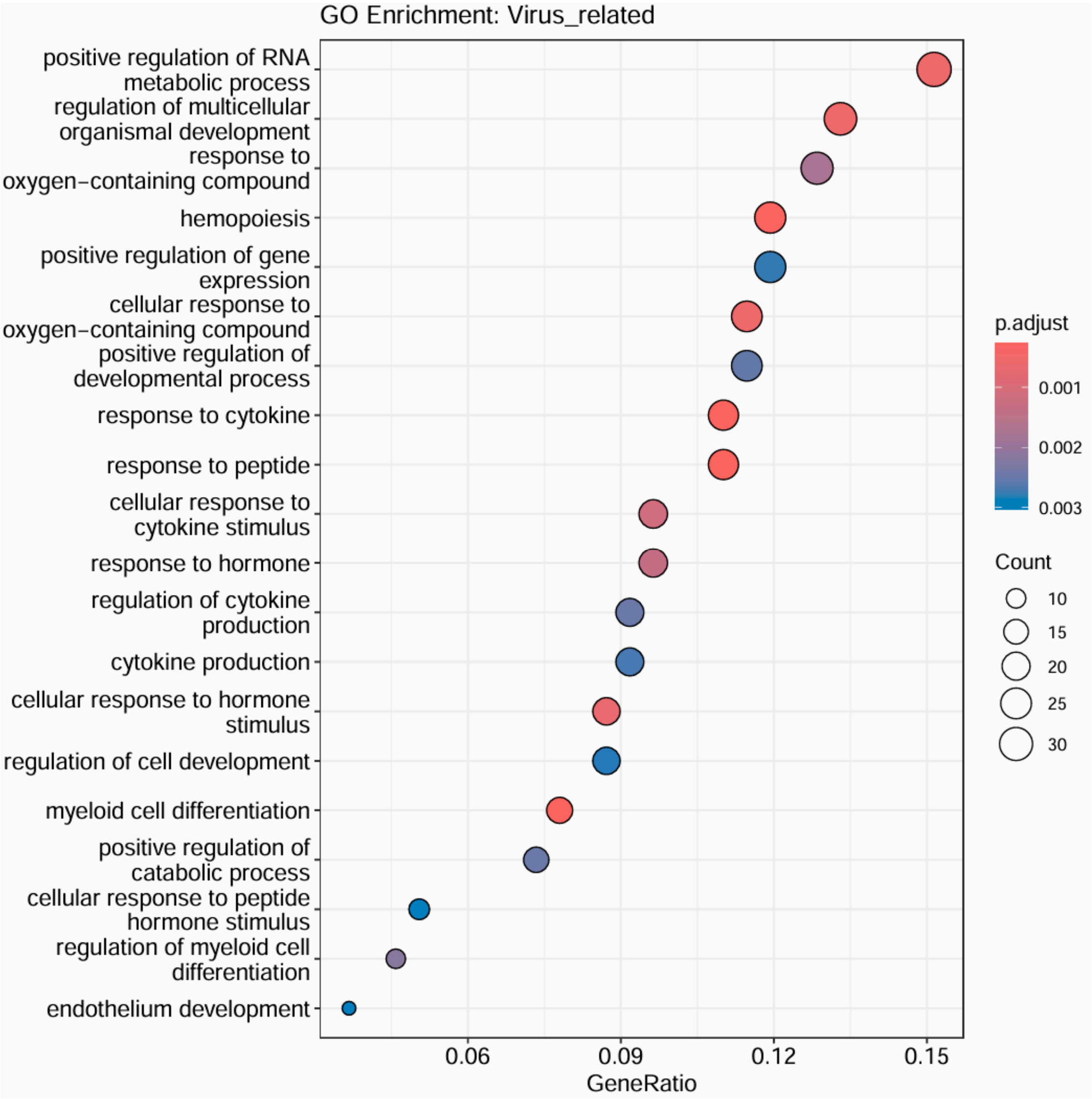

Supplementary Figure S15. GO Enrichment analysis of Netrin pathway positively related host genes

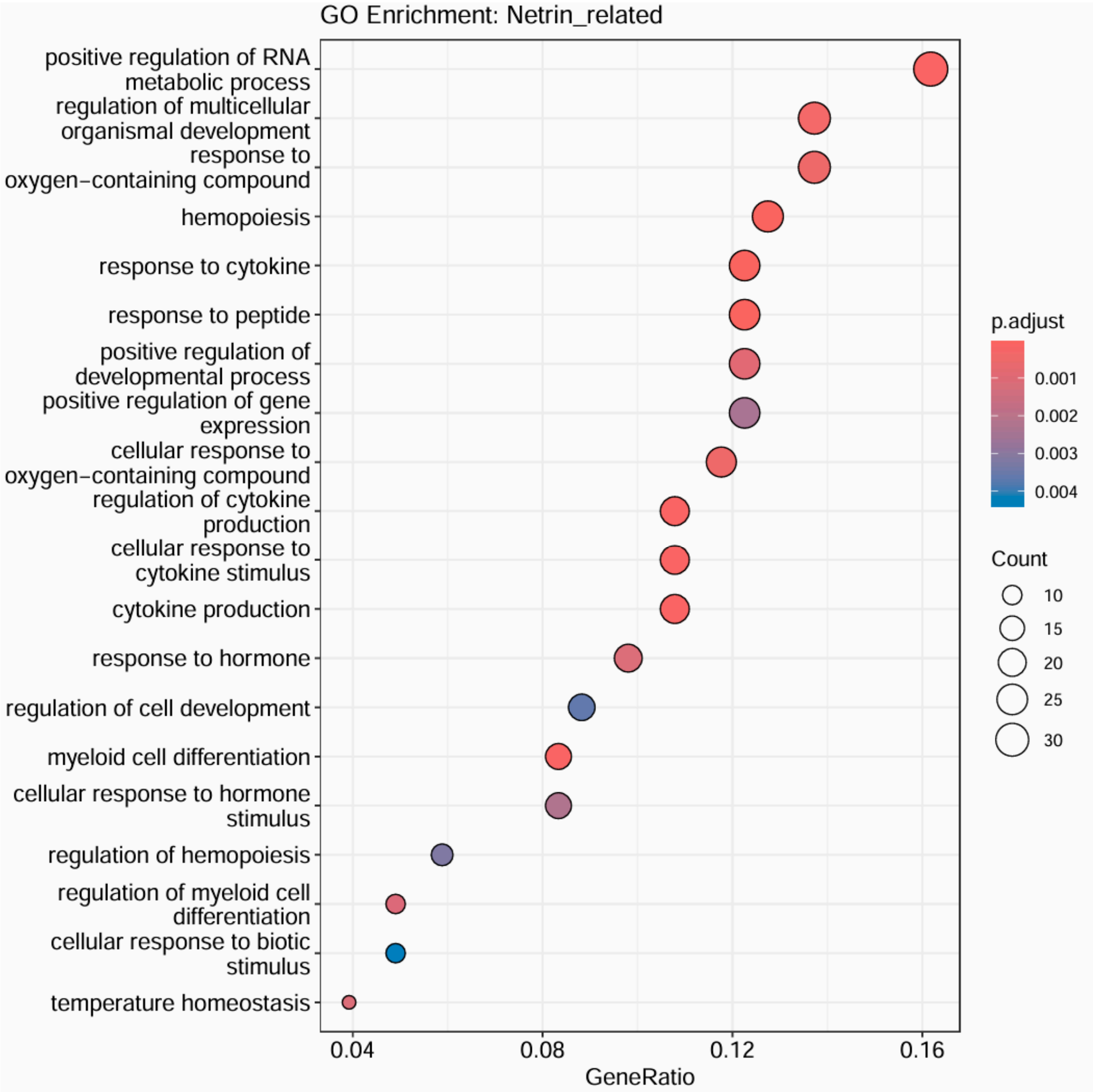

Supplement: Supplementary file 1 [file biology-14-00882-s001.zip › biology-3728407-supplementary-Figures.pdf]
